# Approach to assess the performance of waste management systems towards a circular economy: waste management system development stage concept (WMS-DSC)

**DOI:** 10.1016/j.mex.2022.101634

**Published:** 2022-02-17

**Authors:** Alessio Campitelli, Jan Kannengießer, Liselotte Schebek

**Affiliations:** Technical University of Darmstadt, Institute IWAR, Department of Material Flow Management and Resource Economy, Franziska-Braun-Straße 7 64287 Darmstadt, Germany

**Keywords:** Benchmark, developing countries, leapfrogging, global north, global south, municipal solid waste, holistic, monitoring, CE, Circular economy, CMUR, Circular material use rate, comp., Company, CT, Collection and transport, DR, Diversion rates, DTT, Distance-to-target, ER, Energy recovery, EU, European Union, G, Governance, MSW, Municipal solid waste, mun., Municipal, nat., National, PR, Prevention and reuse, RDF, Refuse-derived fuels, reg., Regional, SDG, Sustainable development goal, SM, Sector and market, UN, United Nations, WD, Waste disposal, WM, Waste management, WMS-DSC, WMS development stage concept, WMSs, Waste management systems, WR, Waste recycling, ZWI, Zero-Waste-Index

## Abstract

In this paper a novel holistic approach to assess the performance of waste management systems (WMSs) is presented. The so called WMS development stage concept (WMS-DSC) can be used by practitioners or decision makers to assess primarily the WMS at the municipal level. The WMS categorization into development stages notably enables a clear identification of symptoms, the causes of possible waste mismanagement and potential measures for improvement. The concept can be used to (i) assess the status quo of a WMS and, based on this, identify possible measures for implementation; (ii) check whether relevant system conditions to implement a specific measure are met; (iii) monitor the progress of a WMS; and (iv) compare the WMSs of different cities. The concept consists of five development stages: stage 1 – absence or lack of essential elements of waste management; stage 2 – reliable collection and improved landfill sites; stage 3 – separate collection and sorting; stage 4 – expansion of the recycling industry; and stage 5 – circular economy (CE), waste as a resource. While stage 1 describes the absence of or a very immature and malfunctioning WMS, stage 5 stands for a fully implemented CE. By equating the highest stage to the objectives that have evolved today globally for a sustainable CE, this concept can be used to identify targets and the most suitable steps for an individual WMS towards a future best practice of CE.

• A holistic approach to assess waste management systems’ performance is presented.
• A benchmarking tool to estimate the circular economy (CE) evolvement in cities.
• Usable for cities both in the Global North and South to identify CE potentials.

## Highlights

• A holistic approach to assess waste management systems’ performance is presented.
• A benchmarking tool to estimate the circular economy (CE) evolvement in cities.
• Usable for cities both in the Global North and South to identify CE potentials.

## Graphical abstract

**Figure:**
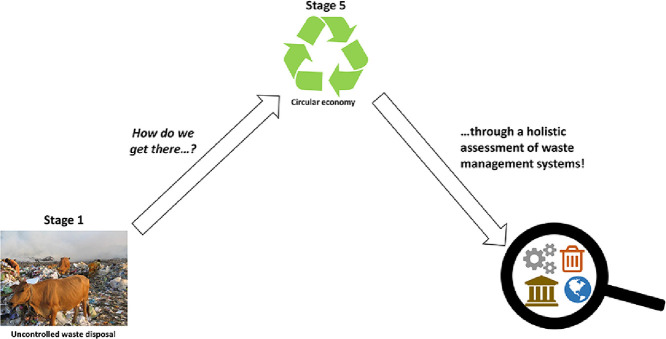

Specifications Table

**Table:** 

**Subject Area:**	Environmental Science
**More specific subject area:**	Waste Management Systems
**Method name:**	Waste management system development stage concept (WMS-DSC)
**Name and reference of original method:**	Not applicable
**Resource availability:**	Not applicable

## Introduction

The assessment of waste management systems (WMSs) is a fundamental step to develop and improve waste management (WM). From a global perspective, the status of WMSs spans an extremely wide area. In many low-income countries, even the most fundamental components of WMS, ensuring health and environmental safety of the population, are partly lacking. In contrast, in high-income countries, WMSs are far developed regarding technology as well as organizational and logistical management and ensure a safe and environmentally sound treatment of waste [17]. Still, also here, WMSs are considered insufficient in view of their contribution to sustainable development, notably the mitigation of climate change and reduction of the demand for primary raw materials. To tackle this challenge, in recent years, the concept of a circular economy (CE) has evolved, notably in the European Union (EU), where it has been adopted as a guiding principle of policy [14]. A CE is “[…] an economic system that is based on business models which replace the ‘end-of-life’ concept with reducing, alternatively reusing, recycling and recovering materials in production/distribution and consumption processes, thus operating at the micro level (products, companies, consumers), meso level (eco-industrial parks) and macro level (city, region, nation and beyond), with the aim to accomplish sustainable development, which implies creating environmental quality, economic prosperity and social equity, to the benefit of current and future generations.” [18]. The idea of CE is meant to reduce waste to a minimum and keep materials and products as long as possible in the economic cycle [19]. Therefore, the vision of CE comprehends the promotion of prevention, reuse and recycling of waste and concurrently the reduction of waste that is being incinerated or landfilled.

Kirchherr et al. [18, p. 229] stated that “[…] CE must be understood as a fundamental systemic change instead of a bit of twisting of the status quo to ensure its impact.” Consequently, this means that to reach a CE status, it is necessary to assess the whole WMS, including all related components. Thus, the assessment of WMSs can be seen as an essential tool to monitor and foster the global efforts towards a CE. In order to achieve this, an assessment methodology has to cover all relevant system components of a WMS (e.g., governance, waste market, collection, recycling and recovery, disposal, prevention and reuse), identifying the determining characteristics of any state and relating these to the identification and successful measures for improvement.

In a review by Campitelli and Schebek [7], 366 peer-reviewed articles assessing WMS in cities and published before May 2019 were analyzed concerning the used assessment methods and the analyzed WMS components. The review shows that mostly, life cycle approaches were used solely (n. 92) or in combination with other methods (n. 54) to analyze WMSs. Fifty-three (53) studies used benchmarking tools and performance indicators for WMS analysis, whereof 21 used them in combination with other methods. Out of the 366 case studies, four studies examined all relevant WMS components simultaneously using surveys [1], a combination of methods such as scenario analysis and performance indicators [15], life cycle approaches in combination with economic models [2] and benchmarking tools combined with surveys [34].

Moreover, to assess the WMS as well as the CE performance of a city, a large amount of data and information are needed. Methods such as life cycle or other sustainability assessment approaches need a great amount of data, which is often lacking in countries of the Global South. Consequently, benchmarking is a suitable method for such cases because, depending on its design, it can work with both qualitative and quantitative information [7], even if data is lacking.

A well-known benchmarking tool for WMS analysis is the “Wasteaware” benchmark tool developed by Wilson et al. [33], which allows the examination of various system components of a WMS (e.g. collection, recycling, disposal, governance). Mendes et al. [21] adapted the management tool Balance Scorecard from [16] to analyze public and private waste organizations in Portugal. Recently, new benchmarking methods have been published, which assess WMSs by defining WM development ranges as sort of maturity levels [13] or development bands [31]. Both methods see the achievement of a CE as the final achievable level of WM development. While Fatimah et al.’s [13] approach focuses more on the analysis of an industry 4.0 based smart CE, the framework of Whiteman et al. [31] was developed to be mainly applied in low- and middle-income countries to promote the WMS development towards a CE. UN-Habitat [29] also developed the Waste Wise Cities Tool, which consists of guidelines on how cities should collect waste related data with the aim of reducing the data gap. Furthermore, it also includes control levels, “ladders”, for different WMS components (collection, disposal, recycling and recovery). This is a diagnostic tool for WMS performance assessment in cities all over the world, but it is mainly meant to support cities from the Global South, where lack of data is a crucial problem that hinders WMS development.

All benchmarking methods stated above can be used for rapid assessment of the WMS performance or for specific parts of a WMS, but they are limited in terms of an in-depth and holistic analysis of the whole WMS performance. Therefore, holistic approaches to WMS assessment are still rare or are usually presented in a simplified way, such that the level of detail and completeness is reduced [7]. Often, WMS components regarding the waste sector and market, prevention and reuse are missing or underrepresented in the assessment as well as issues regarding governance.

This paper presents a novel holistic approach based on the general idea of development stages for the assessment of WMS performance. The approach presented here enables an in-depth analysis of the WMS performance for cities and goes further in comparison with the approaches of Fatimah et al. [13], Whiteman et al. [31] and UN-Habitat [29].

The structuring based on development stages has already been applied in other application areas, e.g. by the European Commission for capacity building [10]. In the context of WM, the German Ministry of Environment defined five technological development stages, starting from the extensive uncontrolled dumping and ending with the status of CE [4]. Striegel [27] described ten fields of action subdivided into five development stages to support low and middle income countries in establishing WMSs [27]. Based on these publications, the general idea of the WMS-DSC was initiated in 2017.

In contrast to the other benchmarking methods, our approach goes further and comprises the following novel and unique features:

• WMS is characterized clearly by components and systems boundaries.
• Every development stage is worked out as a detailed hypothetical scenario for all relevant WMS components (see Table 1), including different dimensions such as governance, economic, social, organizational, environmental and technical aspects [7].

**Table 1:** Overview of the general structure of the WMS-DSC matrix (empty form)

	Stage 1	Stage 2	Stage 3	Stage 4	Stage 5
Governance					
Sector and market					
Collection and transport					
Waste disposal					
Energy recovery					
Waste recycling					
Prevention and reuse
• The performance assessment is expanded by the distance-to-target (DTT) concept, which is a widely used method in life cycle assessment to assess the actual state of a specific issue and relate it to the desired state (target). The distance to the target is normally expressed by a specific value [9]. However, the integration of DTT in WMS assessment concept is still missing. By integrating the DTT concept, it is possible to estimate the quantitative or qualitative distances to a higher development stage, which in this paper are expressed by pre-defined stages.
• Based on the analysis results, measures to improve the WMS can directly be identified by the user of the WMS-DSC, considering the higher stages.
• Interactions between the different WMS (sub)components are visible: firstly, this enables a better understanding of the complex system of WM; secondly, it helps to identify possible causes of waste mismanagement.

## Method details

The approach presented here is called WMS development stage concept (WMS-DSC), and a categorization scheme for characterizing the development stages of WMSs is introduced. It can be used by practitioners or decision makers, who are familiar with the WMS under study, (i) to assess the actual WMS and (ii) to analyze whether the system conditions are met for the introduction of specific (e.g. technological) measures. Besides the analysis of the status quo, it can also be used (iii) to monitor the progress of a WMS and (iv) as a benchmarking tool to compare WMSs, for example of different municipalities. Regardless of the motivation for using the tool, recommendations for action or improvement measures can be derived based on the assessment results and the scenario descriptions. The WMS-DSC is primarily designed to assess the WMSs of cities and municipalities, but the structure of the concept enables also an assessment of WMSs on a regional or national scale. However, by doing this, it must be noted that the holistic character is clearly reduced as some criteria only apply to the municipal level.

The WMS-DSC can generally be used for all WMSs (urban and rural) and is adaptable to countries of the Global South and Global North. The concept is primarily focused on municipal solid waste.

### WMS-DSC approach

The WMS-DSC approach consists of a matrix, in a similar style as the capacity scanning matrix of the European Commission [10]. The WMS-DSC matrix is based on a checklist (see S1 “WMS-DSC simplified Excel tool for assessment” and S2 “Detailed WMS-DSC matrix description”) that frames five development stages for seven WMS components. The general structure of the matrix is shown in Table 1, and the detailed descriptions in Table 2.

**Table 2:** Description of the WMS components adapted according to Campitelli and Schebek [7].

WMS components	Description
**Governance** **“G”**	All measures that are needed to manage, control and regulate WM at the municipal, regional or national level. For example laws, regulations, policy and financial instruments, waste plans, programs, concepts, authorities, and other supervisory bodies, institutions, or WM services. This component is divided into 16 subcomponents assigned to four groups: • **Legislation and other policies (G.1 – G.6):** Duties and targets; laws, regulations and agreements; nomenclature; national WM; regional WM; and municipal WM • **Administration and monitoring (G.7 – G.11):** Control mechanisms; authorization and public participation; quality standards and threshold value; data collection; reporting and evaluation • **Education and research (G.12 – G.14):** Education; research; awareness building • **Occupational safety, health and environmental protection (G.15 – G.16):** Occupational health and safety measures; environmental protection measures

**Sector and market** **“SM”**	All market activities and aspects concerning WM (including recycling markets, import and export of waste, employment); the structuring of the WM sector (including public and private company structures); the integration of the informal sector; financial funding of WM activities and waste projects; trading and broking of waste products or secondary raw materials and fuels. This component is divided into seven subcomponents: • **Sector and Market (SM.1 – SM.7):** Sector development; jobs; informal sector; WMS structure and organization; financial funding; enterprises; recycling market

**Collection and transport** **“CT”**	Measures such as the collection of waste systems (e.g. door-to-door), separation at source of different waste types (MSW; hazardous waste, etc.), and their transportation and storage (e.g. transfer stations) are included here. Also, aspects concerning service providers and collection efficiencies and collection rates are included. This component is divided into eight subcomponents: • **Collection and transport (CT.1 – CT.8):** Waste collection; service provider; collection rates; separate collection; collection of recyclables; waste transport; transfer stations; collection of hazardous wastes

**Waste disposal** **“WD”**	All measures and aspects regarding the disposal of waste (open dumping, open burning, landfilling, and other means of disposal), the different qualities of landfilling, and leachate and landfill gas management are included. This component is divided into five subcomponents: • **Waste disposal (WD.1 – WD.5):** Waste disposal; operational measures; leachate water management; landfill gas management; other means of disposal

**Energy recovery** **“ER”**	Aspects and measures of plants that use waste as a fuel (like the controlled incineration of waste to produce thermal or electrical energy), co-incineration and the conversion of RDFs into energy as a substitute for fossil fuels are described here. This component is divided into three subcomponents: • **Energy recovery (ER.1 – ER.3):** Thermal disposal and energy recovery; incineration plants; energy and raw material recovery

**Waste recycling** **“WR”**	Measures to recycle different wastes, such as composting, fermentation, sorting and other recycling plants (especially material recycling), are described here as well as recycling rates, diversion rates (DR) and the circular material use rate (CMUR). This component is divided into eight subcomponents: • **Waste recycling (WR.1 – WR.8):** Waste recycling; composting, fermentation, sorting and recycling plants; RDFs; recycling of construction and demolition waste; recycling rates; DR and CMUR

**Prevention and reuse** **“PR”**	Measures to prevent waste and promote reuse activities as well as innovative business models, such as sharing and repairing and efforts of companies regarding the optimization of processes and products (product design), are included here. It also comprises indicators such as waste generation and Zero-Waste-Index (ZWI). This component is divided into six subcomponents: • **Prevention and reuse (PR.1 – PR.6):** Prevention; circular business and usage models; product design; process optimization and operational disposal strategies; waste generation; ZWI

The five stages are based on the defined development stages of BMUB [4]:

1^st^ Stage: “Absence or lack of essential elements of waste management”
2^nd^ Stage: “Reliable collection and improved landfill sites”
3^rd^ Stage: “Separate collection and sorting”
4^th^ Stage: “Expansion of the recycling industry”
5^th^ Stage: “Circular economy – waste as a resource”

While stage 1 describes a very immature and poorly functioning WMS, stage 5 is equivalent to a successful CE. The increase of the development stage always implies an improvement in comparison to the lower stage. Every development stage represents a hypothetical scenario. The stage descriptions have their origins in BMUB [4] and Striegel [27], but they have been further modified and described in detail, taking into account additional system components and different literature.

For this purpose, various research articles on WM and CE have been reviewed and included, which are cited in the detailed WMS-DSC (S2). For stage 1, literature mainly dealing with WM problems and challenges, especially in low and middle income countries, was used for the description. The stage descriptions from stage 2 onwards are principally oriented towards the EU's WM evolution regarding directives and guidelines as well as objectives and other WM activities (technological, environmental, etc.). Therefore, the European WM and CE activities and their waste related legislation are used as a benchmark, which was also done in other studies [3,^5^,26]. Moreover, stages 2 and 3 rely mostly on guidebooks on WM for the implementation of waste treatment plants, e.g. from the World Bank, and on findings and experiences from relevant scientific studies. In stage 4, publications illustrating the successful implementation of novel measures and the state of the art are used as basis. In addition, studies and documents with targets and recommendations for action to increase the CE potential, such as the CE Action Plan of the EU [11], were incorporated. For stage 5, which represents a functioning CE (best-case scenario), literature with current CE research topics and with CE visions was taken into account [6,^11^,^13^,^14^, ^22^,31]. Despite the fact that the stages derived from the historical evolvement of the German and European WM and stage 5 contains visions and ideals of a CE, which are also being discussed in the EU, they also contain other visions of a CE society, which are not directly related to Germany or the EU. Consequently, it does not mean that one or more German or European cities were used to describe stage 5. Stage 5 is a fictional best-case scenario, which describes a well-functioning CE. This scenario does not exist in any city yet. It is advantageous to use this concept in any case if the city under study wants to follow the CE definition of the EU or if the CE potentials have to be identified.

All stages have assigned WM driver(s) [32] and main target(s), enabling a clear mapping of the individual stages. The detailed descriptions of all stages are presented as follows:

**1^st^ Stage: “Absence or lack of essential elements of waste management”**

The main driver and target for this stage is the removal of any kind of waste. The waste is not sorted at source and discarded on uncontrolled dumping sites or openly burned. The legal situation is very weak and there are almost no control mechanisms. Lack of responsibilities and unclear structures lead to unregulated, unreliable, ineffective, and expensive waste collection and disposal activities. There is a huge lack of waste-related data or statistics. The informal sector is an essential part of the WM as it operates in waste collection and sorting of recyclables. Occupational safety, health protection, and climate and environmental protection measures are almost non-existent.

*Driver: Removal of waste*

*Main target: Collection and removal of waste from housing areas*

**2^nd^ Stage: “Reliable collection and improved landfill sites”**

In this stage, the protection of humans from the health hazards associated with improper disposal of wastes is becoming more important. The main target of this stage is to stop uncontrolled dumping and open burning and to move towards a controlled disposal of waste. Also, the legal situation is getting more stringent and specific; responsibilities are defined, and control bodies have been implemented. Waste related data collection is introduced to evaluate the actual WM at the municipal, regional, or even national scale. Waste concepts, plans, or programs are becoming more important to make existing WM more transparent and create disposal security. Waste collection has been further expanded, but has not been widely established (especially in rural areas). The financing of WM is getting more stable. Simple recycling plants (e.g. composting plants) are implemented. WM offers great employment potential, but there is a lack of qualified personnel. Health security in the waste sector, urban hygiene, and conditions in the informal sector have been improved. Recycling markets are expanding.

*New driver: Protection of human health*

*Main targets: Stop of uncontrolled dumping and open burning*

**3^rd^ Stage: “Separate collection and sorting”**

This stage is predominantly characterized by the driver “environmental and climate protection”. The reduction of emissions from landfills and its volume are the main targets of this stage. Waste segregation at source, higher collection rates, and the increased use of sorting facilities are the basis of high-quality sorting. Waste collection services are becoming more efficient. Additionally, due to stricter regulations concerning the disposal of waste in sanitary landfills, energy recovery, anaerobic digestion and the use of waste as refuse-derived fuels (RDF) are considered relevant alternatives of waste treatment. Sales markets emerge for the use of RDF and other recycling products. Regulations on the monitoring of emissions released from waste treatment plants have been implemented to reduce emissions from WM. The informal sector has been completely formalized through the formation of responsible umbrella organizations or interest groups or by their integration into private sector enterprises. Extended producer responsibility is introduced to make distributors and manufacturers more accountable for their products after becoming waste. This may lead to a possible formation of dual systems or other take-back concepts as well as efforts in ecological product design. Also, more jobs are being created in the WM sector regarding the construction and operation of treatment plants. An intensified use of automation can lead to higher occupational health and safety in the waste sector. Initiatives and programs to strengthen the environmental awareness regarding waste are conducted.

*New driver: Environmental and climate protection*

*Main targets: Reduction of landfill volume and its emissions*

**4^th^ Stage: “Expansion of the recycling industry”**

First, efforts are made to close material loops not only on a national scale but also on a company scale (including industrial symbiosis). Therefore, material recycling of waste becomes a matter of growing relevance. The main driver of this stage is the effective use of waste as a valuable resource. The primary target is to expand the use of waste as a resource to produce secondary raw materials. The sector is expanding and its importance in the context of resource policy is rising. WM planning is integrated into resource and energy efficiency concepts.

Modern and high-end technologies are used to produce mono fractions with a high quality or to treat wastes properly, in an environmentally friendly and resource-efficient manner. Recovery of fuels from waste through waste-to-energy plants (incineration, fermentation plants, etc.) are alternatives to material recycling. Landfilling of inert and pre-treated waste is still practiced, but landfilling rates of municipal solid waste (MSW) are decreasing. Waste prevention is getting more and more important.

WM services are more often delegated to private providers or to WM associations as well as public-private partnerships. Competition in the waste sector is growing. Qualified personnel, especially in the development, planning, construction, and operation of advanced technologies, are strongly requested.

*New driver: Resource value of waste*

*Main target: Increased use of waste as a resource*

**5^th^ Stage: “Circular economy – waste as a resource”**

Conserving natural resources is the essential driver of this stage. This stage describes visions of the future, considering a successful implementation of a CE. The concept of the CE (including circular bioeconomy) is intended as a fundamental concept of resource management and is put into practice. Objectives such as waste prevention, substitution of fossil resources with bio-based alternatives, and enhancing resource efficiency are pursued to a significant extent. The recirculation of waste materials into the material cycle can be verified by very high recycling rates. The transparent data situation in the field of production and WM in combination with a more extensive use of digitization enables a more efficient and resource-saving economy. The reuse of products and the prevention of waste result in an ongoing trend towards more innovative business models, such as leasing, sharing, and low-packaging trade, which gain a high social standing and are actively used by large proportions of the society and enterprises. The quality of recyclates and their quantitative availability are publicly monitored and reported, so that secondary materials are systematically and extensively used in industry to minimize supply risks and to remain competitive. The practical application of CE makes a significant contribution to a resource-efficient and low-carbon economy, which can be clearly monitored by the respective sustainability indicators.

*New driver: Conserve natural resources*

*Main target: Avoid waste and increase resource efficiency*

By categorizing a WMS into development stages, it is possible to identify symptoms and causes of waste mismanagement and potential measures for improvement. Furthermore, it enables decision makers to identify how the WMS, for example in a city, is structured and how the performance of the WMS is evolving. Therefore, this framework can be used by local actors for self-assessment to detect essential WMS activity fields. This can also happen in cooperation with development partners (e.g. sponsors as the World Bank) to identify the next WMS improvement steps. However, the final introduction of future measures for this sector requires experts in the field of WM, who are responsible for designing the measure(s) implementation as well as verifying the financial affordability of the future implementation.

Due to a missing universally accepted definition for WMSs, a harmonized definition has been derived to include all relevant aspects in the WMS-DSC. A *system* generally consists of boundaries, components, and existing interdependencies between the various components. Moreover, it comprises all technical, organizational, and other resources required for the independent performance of a group of tasks or a function [30]. Furthermore, the WMS meets the requirements to be classified as an *infrastructure system* as well. It is a public good as well as a basic condition for economic activities within a country or across national borders to take place [23]. It is a long-living and capital-intensive system and is highly regulated with a networked nature due to the existing interdependencies and complementarities within the system itself and with other infrastructure systems, for example water, traffic, and energy [20]. It is not a classic network infrastructure, such as traffic, water and energy infrastructures, but WM has networking characteristics (e.g. collection routes, waste treatment spots) [8]. The numerous linkages between the components inside and outside the WMS makes it a very complex system [25].

By combining the above mentioned-issues with the definitions of WM from the amended European Waste Framework Directive 2008/98/EC (Directive (EU) 2018/851) [12] and the United Nations Environment Glossary [28], the definition of the term WMS is as follows:

*A waste management system (WMS) is a system-relevant infrastructure system that has the function of collecting, treating (including sorting) and disposing all types of generated waste. Activities that promote waste prevention and reuse or concern trading and broking of waste or secondary raw materials are also part of the system as well as components regarding all governance (e.g. public authorities, laws, regulations), technical (e.g. treatment technologies), and organizational (e.g. infrastructure, collecting services) aspects and other resources (e.g. qualified labor, financial resources, natural resources) required for this function.*

According to this definition, we here specify a) WMS components and b) system boundaries.

**a) WMS components**

The considered WMS components in the WMS-DSC are based on the WMS components’ definitions in the systematic literature review of Campitelli and Schebek [7] in which seven relevant WMS components were identified. Considering the above WMS definition, the components’ definitions have been further adapted (Table 2).

**b) System boundaries**

In general, when assessing systems, it is of particular relevance to define the system boundaries in which the system to be assessed is located. Depending on the chosen assessment objective, the system boundary can vary. The modular design of the WMS-DSC makes it possible to examine a specific WMS component (e.g., governance only) or political scale (e.g., national level only), but it has to be taken into account that this limits also the holistic nature of the concept.

**Fig. 1:**
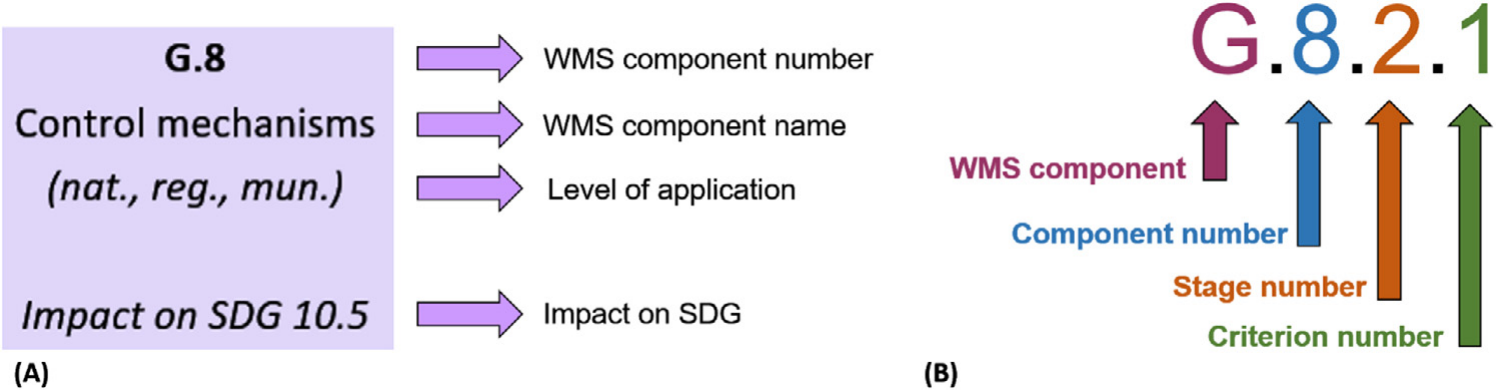
Description of (A) the structure of the subcomponents and (B) the coding of the criteria.

In the detailed WMS-DSC (S2), the subcomponents and their criteria follow a clear structure, which is presented in Fig. 1. The structure is illustrated by the example of the subcomponent G.8 (Fig. 1A) and its criterion G.8.2.1 (Fig. 1B).

Beside the WMS component number and the name, the level of application and, if applicable, the impact on the United Nations Sustainable Development Goals (SDG's) are mentioned for every subcomponent (Fig. 1A). In the WMS-DSC, four levels of application have been identified: national (nat.), regional (reg.), municipal (mun.), and company (comp.). These four levels will support the user in assessing a single criterion. They should make it easier for the user to find the respective information to assess the criterion. For example, there are subcomponents that can be found only at the national level, such as national legislation; similarly, waste collection is mostly applied at the municipal level. Moreover, there are some criteria that can be present at multiple political levels in parallel, such as stakeholder cooperation and environmental protection measures (see Table 3).

**Table 3:** List of WMS (sub)components, including their level of application.

Level of application		National	Regional	Municipal	Company
WMS components	WMS subcomponents				
**Governance**	G.1 Duties and targets	x			
	G.2 Laws, regulations and agreements	x			
	G.3 Nomenclature	x			
	G.4 National level WM	x			
	G.5 Regional level WM		**x**		
	G.6 Municipal level WM			**x**	
	G.7 Stakeholder cooperation	x	**x**	**x**	
	G.8 Control mechanisms	x	**x**	**x**	
	G.9 Authorization and public participation	x	**x**	**x**	
	G.10 Quality standards and threshold values	x			
	G.11 Data collection, reporting and evaluation	x	**x**	**x**	
	G.12 Education	x	**x**	**x**	
	G.13 Research	x	**x**		
	G.14 Awareness building	x	**x**	**x**	
	G.15 Occupational health and safety		**x**	**x**	**x**
	G.16 Environmental protection	x	**x**	**x**	**x**
**Sector and market**	SM.1 Sector development	x	**x**	**x**	**x**
	SM.2 Jobs	x	**x**	**x**	**x**
	SM.3 Informal sector	x	**x**	**x**	
	SM.4 WM system structure and organization	x	**x**	**x**	**x**
	SM.5 Financial funding	x	**x**	**x**	
	SM.6 Enterprises				**x**
	SM.7 Recycling market	x	**x**	**x**	
**Collection and transport**	CT.1 Waste collection			**x**	
	CT.2 Service provider			**x**	
	CT.3 Collection rates			**x**	
	CT.4 Separate collection			**x**	
	CT.5 Collection of recyclables			**x**	
	CT.6 Waste transport			**x**	
	CT.7 Transfer stations			**x**	
	CT.8 Collection of hazardous wastes			**x**	
**Waste disposal**	WD.1 Waste disposal	x	**x**	**x**	
	WD.2 Operational measures		**x**	**x**	
	WD.3 Leachate water management		**x**	**x**	
	WD.4 Landfill gas management		**x**	**x**	
	WD.5 Other means of disposal	x	**x**	**x**	
**Energy recovery**	ER.1 Thermal disposal and energy recovery		**x**	**x**	
	ER.2 Incineration plants		**x**	**x**	
	ER.3 Energy and raw material recovery		**x**	**x**	
**Waste recycling**	WR.1 Waste recycling		**x**	**x**	
	WR.2 Composting		**x**	**x**	
	WR.3 Fermentation		**x**	**x**	
	WR.4 Sorting and recycling plants		**x**	**x**	
	WR.5 Refuse derived fuel		**x**	**x**	
	WR.6 Recycling of construction and demolition waste		**x**	**x**	
	WR.7 Recycling rates	x	**x**	**x**	
	WR.8 Diversion rate and Circular material use rate	x			
**Prevention and reuse**	PR.1 Prevention	x	**x**	**x**	
	PR.2 Circular business and usage models				**x**
	PR.3 Product design				**x**
	PR.4 Process optimization and operational disposal strategies				**x**
	PR.5 Waste generation	x	**x**	**x**	
	PR.6 Zero-waste index	x	**x**	**x**	

In addition to the level of application, the level of influence must also be mentioned here, which differs from the level of application. The level of influence means that a criterion whose level of application is national is also directly influencing the underlying political levels. For example, the data or information to assess G.2. Laws, regulations and agreements can be found at the national scale, but it directly impacts regional and municipal waste policies and legislative frameworks.

All criteria mentioned in Table 3 have an influence on municipal WMS assessment. Consequently, this means that if municipal WMSs are assessed, all subcomponents notwithstanding their level of application have to be considered. For regional WMS assessment, it is necessary to consider subcomponents from the national and regional levels (columns 1 and 2 in Table 3), because of the hierarchical dependency. The situation is different if the national WMS is assessed. Accordingly, only subcomponents that can be applied at the national level (column 1 in Table 3) should be considered. When the company level is the focus of assessment, it should be noted that it can only be applied to a limited extent, because of the small number of existing criteria (column 4 in Table 3). However, the concept may be of interest to companies if WM issues are relevant to the site selection of a company or in order to examine the different sites of multinational companies with regard to the existing WM situation in areas where their facilities are located. In this case, the municipal, regional as well as national conditions are also relevant.

### WMS-DSC use instructions

Principally, the descriptions of the components in the different stages correspond to a checklist. This checklist can be practically used by WM experts or other persons who have an in-depth understanding of the current WMS in the investigated city, for example by administrative or local employees or even citizens’ initiatives. The WMS-DSC consists of an Excel checklist for quick assessment (S1) and a more detailed description (S2) of the assessment criteria in the checklist.

The framework should be applied following six steps:

**Step 1: Definition of objective**

In step 1, the objective(s) of the investigation is/are defined:

i) Assessment of the actual WMS state and identification of suitable measures to introduce, based on the actual WMS conditions
ii) Assessment of existing preconditions to implement specific measures (target state)
iii) Monitoring of progress (Condition: Assessment has been done before, e.g. 5 years ago)
iv) Comparison of different WMSs

Independent of the chosen objective, improvement measures can be derived based on the assessment results.

**Step 2: Definition of the system boundary**

Step 2 defines the system boundary of the assessment. Firstly, the WMS under study has to be selected. As mentioned before, the WMS-DSC is designed to assess the WMS at the municipal level (e.g. city or municipality), but also regional and national WMSs can be assessed as well as WMS in the context of companies. If objective (iv) (see step 1) is chosen, then more WMSs case studies have to be selected.

Depending on the selected objective(s) (step 1), it may be necessary to assess all WMS components (e.g. objective i) or only specific components (e.g. objective ii). Therefore, if objective (i) is selected, then it is recommended that all seven WMS components be assessed. If objective (ii) is chosen, then the implementation measure must be defined at this point. Furthermore, the needed preconditions have to be marked in the WMS-DSC to define the “target state”. For example, if the conditions for the implementation of a composting plant is to be examined, it may not be necessary to assess the WMS components ER.1 to ER. 3 regarding energy recovery.

**Step 3: Assessment of the case study**

When assessing a WMS for the first time, it makes sense to assess stage 1 vertically, i.e. all seven components from top to bottom. After stage 1 has been fully assessed, from stage 2 onwards, it is recommended to assess the components horizontally, i.e. from left to right.

In the Excel checklist (S1), the user can choose in the dropdown list if the single criterion is **m** = met, **pm** = partially met, **nm** = not met, or **na** = not available. “Not available” has to be chosen if the geographical circumstances do not enable it (e.g. WD.5.1, waste burning or dumping on high seas and coastal waters, would be obsolete if the city or the nation is not near a high sea or coast), if energy recovery (incineration) is not desired by the city/nation and will not be promoted, or if no data is available (e.g. missing recycling rates). If certain data (e.g. recycling rates, indices) are only available at the national level and not at the municipal level, the national data can be used, but this must be indicated somewhere in the assessment to increase transparency and traceability.

The criteria are as far as possible formulated in a positive way. Conversely, in the best case, all criteria in stage 1 are ticked as “not met,” and from stage 2 onwards, they are ticked as “met”. In general, if a stage 2 criterion is ticked, then, where applicable, the corresponding counterpart in stage 1 should be ticked as “not met”.

Some subcomponents’ criteria are underlined. The underlined criteria represent important milestones that are essential to building a WMS or even a CE. At the end of the assessment, these milestones help to identify essential recommendations for action.

Moreover, some criteria need conditions that are not included in the same subcomponent. If this is the case, the required criteria have been added in brackets; see the following example: “SM.4.3.2: Companies specialized in waste recycling/disposal need a specific certification (G.8.3.4) to work in WM”. The required certification is defined in the WMS component G.8.3.3 (“Introduction of regulated and recognized certification of specialized WM companies”). This means that if the system boundary is set in a way to include only specific WMS components, it is necessary for this case to include the interactions (dependencies), which are part of an excluded WMS component.

**Step 4: Stage determination of subcomponents**

**Fig. 2:**
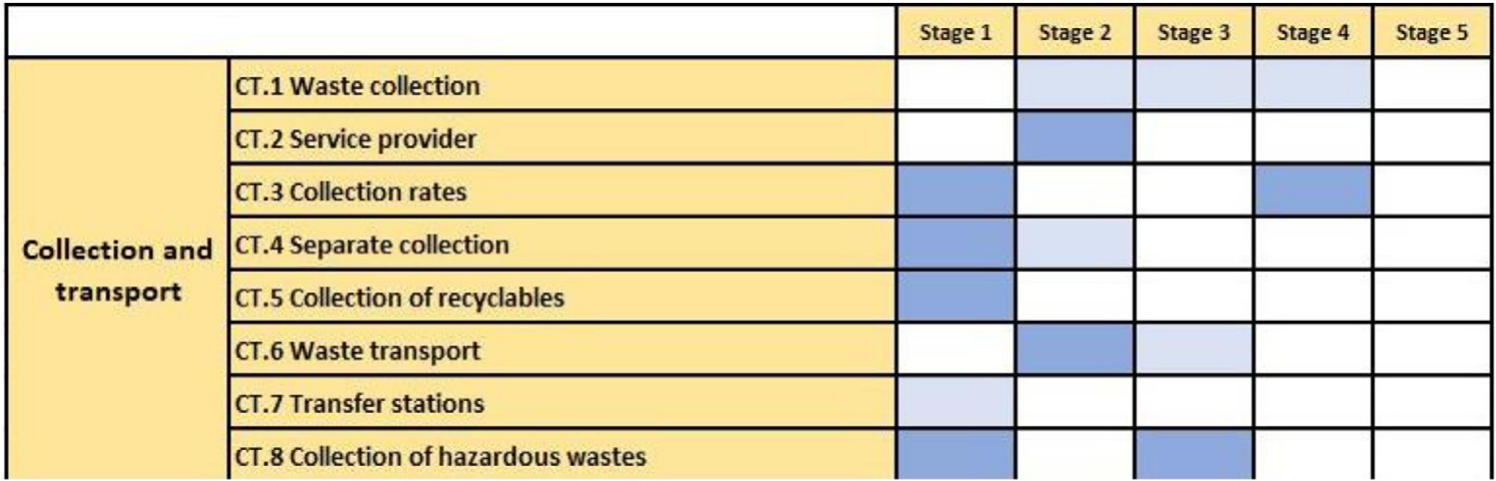
Example of the graphical presentation of the analysis results for the “Collection and Transport” subcomponent.

An exact determination of the stage for a subcomponent is possible, when all characteristics of the stage are ticked. Therefore, for a subcomponent to be fully in stage 3, all criteria of stage 3 have to be met; otherwise, it is only partly in stage 3. The results of the analysis can be directly visualized in S1 in the Excel map “results overview”. In order to provide a consistent visualization of the results table (see Fig. 2), users are advised to do the following:

If the criteria of a stage-related subcomponent....

...are completely “met,” then the fields are marked dark blue.

...are completely “not met,” then the fields are white.

...all other combinations are interpreted as “partly met” and are, therefore, marked with light blue.

If a subcomponent's criteria are met at different stages, then an exact determination is not possible. In this case, the range has to be mentioned (e.g. “CT.3 is between stage 1 and stage 4”). If a WMS component extends over several stages, this shows that the development of the WMS component is ongoing. Therefore, only the criteria that are “met” are considered to define the stages and, finally, the range.

However, this assessment can also be useful in identifying the additional criteria that are necessary to move to the next stage. Considering the DTT principle, in this case, the distance is determined by the stages, and the main target is to achieve stage 5, which is the best-case scenario. If objective (ii) is investigated, then the target is equal to the implementation measure (see step 2).

Subcomponents are not always described consistently in all stages. In some cases, certain criteria are only present at stage 2 or higher (e.g. G.2.3.5) or only go up to stage 2 as the maximum (e.g. G.4.2.1 and G.4.2.2). This means that not every subcomponent can necessarily reach stage 1 or stage 5.

**Step 5: Determination of improvement measures**

Depending on the objective selected in step 1, different result types can be generated. They may be very detailed, involving the identification of specific measures for further implementation (e.g. introduction of separation at source), or more abstract, such as the identification of possible WM activity fields (e.g. promoting material recycling). Depending on the assessment, a large number of measures may result. In order to maintain a clear overview, we advise users to divide the measures into clusters. These clusters may be subcomponent-related. But due to the existing interdependencies between the different subcomponents, it would also make sense to cluster measures that are topic-related (e.g. “Promote recycling,” “Ensure area-wide collection,” “Ensure disposal security”) or aligned to the SDGs.

Furthermore, if different cities or municipalities in a country are assessed, similarities and differences can be identified from the respective city-specific results. An upscaling of the city-related WMS conditions at the national level can be realized if the case studies examined are representative of the country. This analysis permits the drawing of conclusions regarding whether the WM in a country is evolving homogeneously or whether a big diversity has been identified. Finally, by equating stage 5 to a well-functioning CE, with this concept, the CE potential can be estimated and measures derived on how to exploit existing potentials.

**Step 6: Assignment to the United Nations Sustainable Development Goals *(optional step)***

Step 6 is an optional step that can be taken if an assessment of the WMS concerning the SDGs is desired. In the detailed assessment concept (S2), the corresponding SDGs (see example in Fig. 1A) have been assigned to the respective subcomponents where possible. The SDGs and the assigned WMS-DSC subcomponents are listed in S3.

### The use of the WMS-DSC for cities of the Global North and Global South

The descriptions of the stages from 1 to 4 mainly leaned on WM developments in the European area, but developments in low and middle income countries are also taken into account, mostly in the stage descriptions 1 to 3. As mentioned before, the final stage 5 describes a fictional best-case scenario of a well-functioning CE. Therefore, even if a city of a high income country has a good performance (e.g. stage 4) in the subcomponents of governance, at the same time, it may have a weak performance in component prevention and reuse. For cities of the Global South, it works in the same way. They may have good performances regarding waste generation but have great optimization potentials concerning waste disposal. This concept allows the rewarding of aspects that are good or successful in a WMS with a higher stage assignment.

**Fig. 3:**
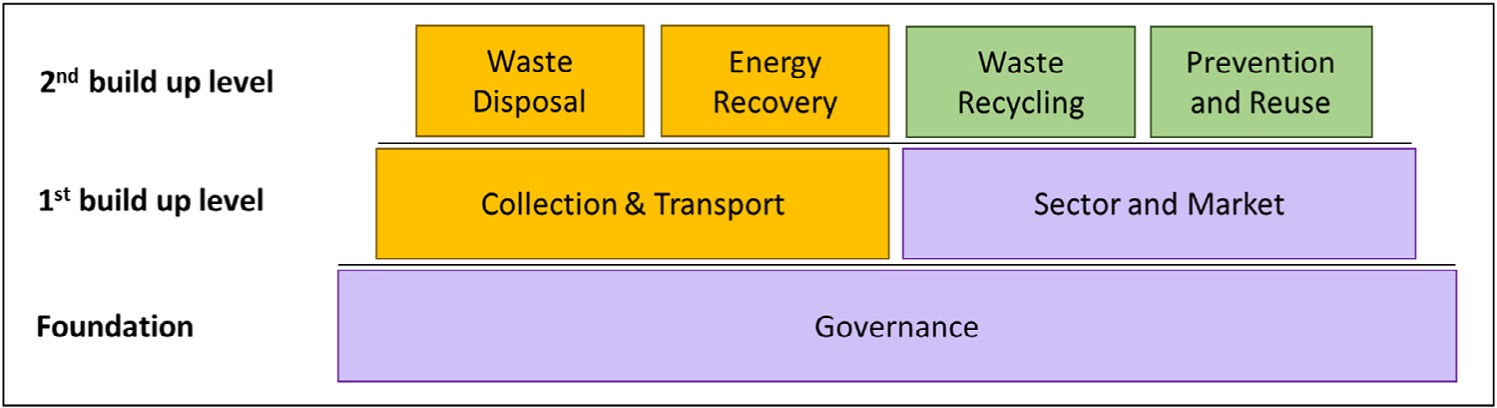
Dependencies between the different WMS components.

When using the concept, it should be noted that the financial barriers for cities in the Global South are greater than those of cities in the Global North, so funding, e.g. from development banks or international alliances, is required. Regarding the CE visions that are described in stage 5, the WM costs may rise with increasing stage. This surely depends on the stages that are targeted (e.g. stage 3 or stage 5) and what infrastructures are already in place. Consequently, implementing certain technologies may not yet be an option for specific cities in the Global South. However, it is definitely important to know the potentials on which the city can build on. It should be highlighted that the vision of a CE does not only mean technological change but also social change, which is why it may take a considerable amount of time, depending on the country, until stage 5 is achieved. It is also important to mention that the concept is independent of time, i.e. the stages of the individual components do not necessarily have to be reached at the same time, since components may be dependent on one another. However, it must be emphasized that, from a systemic point of view, governance must be seen as the foundation, collection and transport as well as sector and market as the first build up level and the other components as the second build up level concerning their dependencies (see Fig. 3).

On the one hand, the concept can support cities to identify necessary measures and to better describe the desired future steps in WM development. On the other hand, the concept may be used also by development banks and other funding agencies to identify funding targets and milestones of projects as well as to review them after approval.

## Conclusion and Outlook

The concept presented in this paper was designed to meet two requirements: First, to represent the extensive complex system of WM as holistic as possible. Second, to present it as simple as possible, so that it can also be used by non-experts, who however have a good knowledge of the WMS under study. Since the concept consists mostly of qualitative criteria, an unequivocal selection is not always possible because it allows a considerable amount of room for interpretation by the WMS-DSC user. This must be taken into account in every assessment case.

In summary the WMS-DSC provides a comprehensive concept, displaying the most relevant but certainly not all existing interactions between the various criteria. But where possible, the relevant interactions have been interlinked throughout the concept. However, it has no claim to completeness, because of the high complexity of the system and the rapid development in this area. The concept has already been tested for the city of Marrakech (Morocco) and for the Seychelles. The results of the case study of Marrakech will be published in a separate publication.

This categorization approach can also be used for educational purpose in universities to facilitate the understanding of WMSs and the existing interconnections between the various WMS components. This is a great chance, also, to raise awareness regarding WM. Furthermore, due to existing educational projects in Morocco and Ivory Coast, the authors are planning to translate this WMS-DSC into different languages (e.g. French), so that it can be used by more countries, for example francophone states.

Due to the fact that the WMS-DSC method enables an in-depth analysis of the WMS and CE performance of a city, it could also be applied after the usage of the rapid assessment approaches of Fatimah et al [13]., Whiteman et al [31]. and UN-Habitat [29] to get the big picture of the WMS performance.

There is great research potential to create such development stage concepts for other areas. In a modified form, the concept can be created for other infrastructure systems (e.g. wastewater, energy, and transport) as well as for industrial sectors (textile processing, agriculture, etc.). There is a chance that these concepts could be used as a basis in the context of Water-Soil-Waste Nexus [24].

## Declaration of Competing Interest

The authors declare that they have no known competing financial interests or personal relationships that could have appeared to influence the work reported in this paper.

## Appendix Supplementary materials

S1: WMS-DSC simplified Excel tool for assessment Image, application 1

S2: Detailed WMS-DSC matrix description Image, application 2

S3: Overview of used SDGs and their assigned WMS-DSC subcomponents Image, application 3
