# Supplementary material for: Approach to assess the performance of waste management systems towards a circular economy: waste management system development stage concept (WMS-DSC)
Source: MethodsX. 2022 Feb 17;9:101634. doi: 10.1016/j.mex.2022.101634 (PMC8886049; doi:10.1016/j.mex.2022.101634)
Supplement: Supplementary file 2 — S2: Detailed WMS-DSC matrix description [file mmc2.docx]

**Approach to Assess the Performance of Waste Management Systems towards a Circular Economy: Waste Management System Development Stage Concept (WMS-DSC)**

#### Abbreviations:

C&D Construction and demolition

CE Circular economy

CMUR Circular material use rate

Comp. Company level

DR Diversion rate

EOL end-of-life

EPR Extended producer responsibility

FE ferrous materials

FGC Flue gas cleaning

GDP Gross Domestic Product

GHG Greenhouse gas

GPS [Global Positioning System](https://de.wikipedia.org/wiki/Global_Positioning_System)

MIS Management information systems

Mun. Municipal level

Nat. National level

NF non-ferrous materials

NGO Non-Governmental Organization

OHSM Occupational health and safety measures

PPP Public-Private-Partnership

Reg. Regional level

R&D Research and development

RDFs Refuse-derived fuels

SDG Sustainable development goal

WM Waste management

ZWI Zero-waste index

|  | **Stage 1**  **Absence or lack of essential elements of WM** | **Stage 2**  **Reliable collection and improved landfill sites** | **Stage 3**  **Separate collection and sorting** | **Stage 4**  **Expansion of the recycling industry** | **Stage 5**  **Circular economy - waste as a resource** |
| --- | --- | --- | --- | --- | --- |
| **Driver(s)** | **Removal of waste** | **Protection of human health** | **Environmental and climate protection** | **Resource value of waste** | **Conserve natural resources** |
| **Target(s)** | **Collection and removal of waste from housing areas** | **Stop of uncontrolled dumping and open burning** | **Reduction of landfill volume and its emissions** | **Increased use of waste as a resource** | **Avoid waste and increase resource efficiency** |
| **Governance – Legislation and other policies I (G.1 – G.3)** | | | | | |
| **G.1**  Duties and targets  *(nat.)* | 1. National [waste management](#WM) (WM) targets are missing. | 1. Preliminary **WM targets** are defined in legislation. | 1. Environmental and climate protection aspects are included in targets formulation. 2. Time dependent targets to develop sustainable WM systems exist at all political levels^[[1]](#footnote-2)^ [1]. 3. WM targets are reviewed regularly [1] and their (non- )achievement is communicated openly. | 1. WM targets are formulated based on Sustainable Development Goals (SDGs). 2. Formulation of targets for CE and [Zero-Waste](#zerowaste) targets [2]. 3. The wording changes from WM to [circular economy (CE)](#CE); Wastes = Resources [3]. | 1. Achievement of ambitious targets regarding CE and waste avoidance. |
|  |  | 1. **Waste disposal is a state responsibility.** |  |  |  |
|  | 1. A defined waste hierarchy is missing. |  | 1. A **5-stage waste hierarchy** has been introduced:  1. prevention - 2. preparation for reuse - 3. material recovery - 4. [energy recovery](#energy_recovery) - 5. disposal. |  |  |
| **G.2**  Laws, Regulations and Agreements *(nat.)*  *Impact on SDG 12.1, 12.4, 12.7 SDG 12.c.* | 1. A national law on [waste disposal](#waste_disposal) is missing or in preparation [4]. | 1. Preliminary waste law^[[2]](#footnote-3)^ regulating waste disposal at national level is in force [4,5].  - Formulation of definitions for “wastes” and respective types of waste. - Responsibility and role of different [stakeholders in WM](#stakeholder_WM) are identified [1]. - Sanctions for non-compliance with legal regulations are anchored in waste laws. | 1. The **regulatory framework for** [**WM**](#WM) consists of comprehensive waste laws^[[3]](#footnote-4)^ and associated directives (regarding waste streams^[[4]](#footnote-5)^ and treatment plants^[[5]](#footnote-6)^) [2].  - Mandatory recyclables collection from households. - Substance or product bans. - Ban of combined disposal of hazardous and non-[hazardous waste](#hazardous_waste) [1]. | 1. Laws and directives are constantly developed and tightened (max. every 5 years) to promote [CE](#CE)^[[6]](#footnote-7)^, remove barriers [8] and respond to (inter-)national challenges [1].  - Mandatory recyclables collection from businesses. - Financial incentives and eco-labelling to promote sustainable products and processes [9]. - Minimum use rates for recyclates in product manufacturing [10]. - Right to repair^^[[7]](#footnote-8)^^ and further incentives^^[[8]](#footnote-9)^^ are defined to promote CE. | 1. CE is promoted more by market drivers than by legal regulations. |
|  |  | 1. Clear guidelines for local authorities specify how waste laws and policies shall be implemented [11]. | 1. Technical instructions and implementation regulations are legally binding [4]. 2. Media related laws or ordinances (e.g., soil, water protection, air pollution control) enter into force. | 1. Legal areas^[[9]](#footnote-10)^ are more closely interlinked to counteract illegal [export](#waste_export) or import of wastes [4]. |  |
|  |  |  | 1. All WM related international agreements^^[[10]](#footnote-11)^^ are ratified. | 1. Duties from ratified international conventions are fulfilled. |  |
|  |  |  |  | 1. In public procurement, at least 50 % are ecologically efficient and environmentally friendly products [12,13]. | 1. In public procurement, at least 80 % are ecologically efficient and environmentally friendly products. |
|  |  |  | 1. **[Extended producer responsibility (EPR)](#EPR)** is introduced [11]. | 1. Through stewardship obligations and EPR, environmental policy transfers more responsibility^[[11]](#footnote-12)^ (financial or organizational) to manufacturers [7]. 2. An ecological evaluation of license fees in the context of EPR (e.g., for packaging) is implemented [14]. 3. EPR is extended beyond national borders along global supply chains for all [life cycle](#LCA) phases (cradle to grave) [7]. | 1. Cross-border EPR enables promising, fair and enforceable distribution of responsibility along global supply chains [7]. |
| **G.3**  Nomenclature  *(nat.)* | 1. Lack of a legally established and standardized nomenclature for waste. | 1. An initial simple nomenclature for certain types of wastes (organics, residual waste, plastics, metals, glass) is regulated by law [4]. | 1. A detailed **nomenclature^[[12]](#footnote-13)^ for waste types** is regulated by law with the differentiation between non-hazardous and [hazardous waste](#hazardous_waste) [4]. | 1. The nomenclature is updated regularly in order to directly take into account new types of waste as well as new state of the art industrial processes and technologies[7]. | 1. (Supra-)National collection of waste data enables a more targeted implementation of measures. |

|  | **Stage 1**  **Absence or lack of essential elements of WM** | **Stage 2**  **Reliable collection and improved landfill sites** | **Stage 3**  **Separate collection and sorting** | **Stage 4**  **Expansion of the** [**recycling**](#recycling) **industry** | **Stage 5**  **Circular economy - waste as a resource** |
| --- | --- | --- | --- | --- | --- |
| **Governance – Legislation and other policies II (G.3 – G.6)** | | | | | |
| **G.4**  National level [WM](#WM)  *(nat.)* | 1. A central body responsible for the development and implementation of WM strategies/policies is missing. | 1. Existence of a **central body** (e.g. ministry of environment) responsible for the development and implementation of WM strategies/policies [1,11]. 2. **National regulatory authority** exists. |  | | |
|  | 1. A national [WM program](#WM_Program) that specifies the development of the waste sector by means of specific measures is lacking. | 1. A **national WM program** exists. | 1. **National** [**waste prevention programs**](#Wasteprevention_programs) (incl. targets and measures) are published. | 1. WM programs, strategies and concepts are revised and specified regularly (max. 5 years) focusing more on climate and resource protection aspects [4]. |  |
|  |  | 1. A **national** [**WM plan**](#WM_plan) exists [5,11]. |  |  |  |
|  |  | 1. Concepts for safe disposal of hospital waste exist. | 1. Concepts for proper disposal or [recycling](#recycling) of mining, construction, forestry, agricultural and [hazardous waste](#hazardous_waste) (industrial sludge, chemical waste, etc.) exist [4]. |  |  |
|  |  | | | 1. Government is increasingly involved in attempts to realize globally led collective actions for sustainable resource management [9]. | 1. The involvement of the government contributes to ensure the proper disposal of waste nationally and combat global environmental problems to a great extent. |
|  |  |  |  | 1. Government implements measures to combat global problems that are related to improper disposal (e.g., marine and space debris, microplastics). |  |
|  | 1. Lack or fragile funding [4] for WM activities [16]. 2. Lack of national funding to improve WM systems on regional or municipal level [17,18]. | 1. A WM budget is set in the federal budget, but it is not guaranteed [16]. 2. National government grants financial aid to regions or municipalities [19]. | 1. **A guaranteed WM budget is set in the federal budget [16], which is sufficient to promote WM activities at all political levels.** |  | |
| **G.5***  Regional level [WM](#WM)^[[13]](#footnote-14)^  *(reg.)*  **(if applicable)*  *Impact on SDG 11.b* | 1. WM functions are dispersed across the regional administration [17]; responsibilities are unclear. | 1. A **central body** (e.g., a ministry of environment) developing and implementing WM strategies or policies and coordinating [waste disposal](#waste_disposal) and recovery activities exists [1,5,11]. | 1. Regional responsibilities regarding waste disposal and [recycling](#recycling) are clear and transparent. 2. **Interdisciplinary cooperation** with other authorities (e.g., spatial planning) exist [4]. 3. Effective mechanisms for siting landfills and other [waste treatment](#waste_treatment) facilities are in place [11]. |  | 1. Regional efforts regarding [CE](#CE) lead to the rise of several circular cities. |
|  | 1. A regional WM law and plan is missing or in preparation. | 1. A **regional waste law** is in force. 2. A simplified **regional** [**WM plan**](#WM_plan) is published. | 1. Regional waste laws are adapted to revised national waste laws (G.2.3.1) 2. A detailed regional WM plan is in place. 3. **Regional** [**waste prevention programs**](#Wasteprevention_programs) are published. | 1. Regional WM laws, plans, strategies and concepts are revised and concretized regularly (max. 5 years) focusing more on climate and resource protection aspects [4]. |  |
|  |  | 1. Regional concepts for the proper disposal of hospital wastes are published. (G.4.2.5) | 1. Regional concepts for mining, construction, forestry, agricultural and [hazardous waste](#hazardous_waste) exist [4]. | 1. Regional plans exist ensuring sound waste disposal in catastrophic events (natural disasters, pandemics, crashes, attacks, war, etc.) [20]. |  |
|  | 1. Due to the lack of or fragile funding basis [4], WM activities are reduced to a minimum. | 1. A more **stable regional funding base** [4] for cost recovery of WM activities is established^[[14]](#footnote-15)^ [17]. | 1. Budget for WM is sufficient to further optimize it at regional and municipal level. | 1. Supra-regional activities promoting [CE](#CE) are implemented. |  |
| **G.6**  Municipal level [WM](#WM)  *(mun.)*  *Impact on SDG 11.b* | 1. Lack of jurisdiction and authority competences of the municipality to plan, finance and operate WM system or to outsource tasks to e.g. private firms [16]. 2. WM functions are spread across different municipal institutions [16–18]; responsibilities are unclear. | 1. **Municipality has the jurisdiction and authority** to plan, finance and operate its WM system or to outsource tasks [5,16]. 2. An **independent WM authority** is established to ensure municipal WM [1]; some functions are still spread across other municipal institutions [18]. 3. Emerging of inter-municipal cooperation’s to carry out WM tasks more efficiently^[[15]](#footnote-16)^ [5,11,21]. 4. Institutionalization of waste counseling for households commercial operators [4]. | 1. **Institutional coherence** [11] in WM is communicated clearly and transparently and publicly available. 2. Cooperation between WM and other municipal service sectors (wastewater management, transport planning, etc.) exist in city management [5]. 3. Public WM companies and authorities use complaints mechanisms [11,17]. | 1. Municipality strives to become a "[Circular City](#CircularCity)“[22]. | 1. The preconditions for achieving the status of "Circular City" [20] are in place. |
|  | 1. A [municipal waste](#municipal_Waste) [bylaw](#waste_bylaw) and [WM concept](#WM_concepts) is missing or in preparation. | 1. A **municipal waste bylaw** is in force. 2. A **municipal** [**WM concept**](#WM_concepts) is publicly available. | 1. Waste bylaws are adapted to revised national (G.2.3.1) and regional waste laws (G.5.3.4). 2. **A municipal** [**waste prevention program**](#Wasteprevention_programs) (incl. targets and measures) is published. | 1. Municipal WM bylaws and concepts are revised regularly (max. 5 years) focusing on climate and resource aspects [4]. 2. Municipal plans exist ensuring sound waste disposal in catastrophic events (natural disasters, war, etc.) [20]. |  |
|  | 1. Due to lack or fragile funding base and high dependence on funds from higher levels (e.g. national government) [4,16–18,23], WM activities are reduced to a minimum. 2. Uneconomic [waste collection](#waste_collection) and disposal [5,18]. | 1. A **stable municipal funding base** [4] for WM activities exists^[[16]](#footnote-17)^ [17,18]. 2. Budgeting and cost accounting methods are used to determine WM costs [18]. | 1. Municipal WM budget is sufficient. | 1. Activities promoting [CE](#CE) in cooperation with foreign municipalities exist. |  |

|  | **Stage 1**  **Absence or lack of essential elements of WM** | **Stage 2**  **Reliable collection and improved landfill sites** | **Stage 3**  **Separate collection and sorting** | **Stage 4**  **Expansion of the recycling industry** | **Stage 5**  **Circular economy - waste as a resource** |
| --- | --- | --- | --- | --- | --- |
| **Governance – Administration and monitoring (G.7 – G.11)** | | | | | |
| **G.7**  [Stakeholder](#stakeholder) cooperation  *(nat., reg., mun.)* | 1. Interaction opportunities between the public sector and other relevant [WM](#WM) stakeholders are missing [16]. | 1. First platforms, committees emerge to promote exchange between the public sector and other relevant WM stakeholders. | 1. Several **stakeholder platforms and committees** exist and are essential for the exchange between the public sector and other relevant WM stakeholders. | 1. Competency centers are established to improve the exchange between relevant WM stakeholders [14]. | 1. Innovative digital exchange formats improve the flow and exchange of information between WM stakeholders. |
| **G.8**  Control mechanisms  *(nat., reg., mun.)*  *Impact on SDG 10.5* | 1. Lack of governmental bodies which monitor and control [WM](#WM) activities. | 1. **National governmental control bodies** for monitoring of WM activities exist. 2. **Regional councils** exist for the lawful enforcement of WM activities on a regional level. | 1. **Certification and accreditation** bodies exist, which are responsible for conformity assessments of e.g., products, persons or bodies. 2. Local authorities monitor activities of firms performing municipal WM functions [5]. | 1. Successful transfer of WM tasks to private and [public-private-partnership (PPP](#PPP)) companies [18] due to skilled supervisory institutions. | 1. Reduction of state regulation [4] due to functioning self-regulation and compliance with existing rules by business and society [24]. |
|  |  |  | 1. Introduction of regulated and recognized **certification of specialized WM companies.** | 1. Definition of nationally standardized requirements for expert qualification and for inspections performance [14]. 2. Regular training of experts is established [14] |  |
|  |  | 1. Monitoring of compliance with legal requirements occurs selectively. | 1. **Stricter controls** are established^[[17]](#footnote-18)^ (G.2.3.1) [4]. 2. State controls are carried out selectively to inspect labor and working conditions. | 1. State controls increase to verify the compliance of labor and worker protection measures. | 1. Control mechanisms work well to identify and sanction illegal behavior and activities in CE. |
|  |  |  | 1. Transport controls of imported and [exported waste](#waste_export) and recycled materials take place. | 1. Regular transport inspections for waste and [recycling](#recycling) materials increase detection of illegal disposal routes. |  |
|  |  | 1. Monitoring of the construction, operation and aftercare of [landfills](#landfill) is carried out selectively. | 1. Monitoring of all landfills (construction, operation and aftercare) and [waste treatment](#waste_treatment) plants takes place. | 1. Strict monitoring of all emissions from landfills and other treatment plants. |  |
|  |  | 1. Despite possible sanctions (G.2.2.1), they are rarely imposed in case of legal violations in WM context. | 1. Sanctioning in case of legal violations is increasing. | 1. Sanctioning has effect and incorrect practices in [CE](#CE) decrease. |  |
|  |  | 1. Control mechanisms to limit corruption and monopoles are introduced but barely applied. | 1. Further development of anti-corruption mechanisms lead to an increased detection of corruption [25]. | 1. Monopolies in CE are regulated [23]. | 1. Corruption is almost contained due to functioning control mechanisms. 2. Corporate monopolies in the WM sector are prevented [5]. |
| **G.9**  Authorization and public participation  *(nat., reg., mun.)* | 1. There is a lack of regulated and systematic official authorization procedures. 2. Public participation is missing in authorization procedures. | 1. **Official authorization procedures** for [WM](#WM) disposal and recovery facilities are implemented. 2. Public participation is included in authorization procedures. | 1. Public participation is mandatory in authorization procedures for large-scale WM plants. 2. Public participation in political WM decisions [24]. | 1. Recognized NGOs can participate in participation and hearing procedures [4]. 2. Public participation in political decision-making processes regarding the [CE](#CE) is desired and made possible [26]. | 1. Full transparency of authorization procedures is guaranteed [4]. |
| **G.10**  Quality standards and threshold values  *(nat.)* | 1. Quality standards and emission limits for waste and treatment disposal plants are lacking. | 1. Quality standards and emission limits for [waste disposal](#waste_disposal) and treatment plants exist. | 1. Permission requirements for disposal and treatment plants are tightened (G.2.3.1). 2. **Emission limits^[[18]](#footnote-19)^ and quality standards are uniform and nationally coordinated**. | 1. Emission limits and quality standards for disposal and treatment plants are updated and tightened regularly (max. every 5 years). | 1. Emission limits and reference values for disposal facilities and secondary raw materials and fuels are tightened in an extent that environmental risks are reduced to a minimum. |
|  | 1. Quality standards (incl. pollutant limits) for secondary materials are lacking. | 1. Quality standards and pollutant limits for certain secondary materials exist. 2. Institutions / associations emerge, who promote unified standards for secondary materials and fuels. | 1. **Introduction of standardized national quality standards for certain secondary materials** (e.g., plastics, glass, compost) and substitute fuels. | 1. Expansion of and tighten quality standards increases the quality of secondary materials and refuse derived fuels ([RDFs](#RDF)). |  |
| **G.11**  Data collection, reporting and evaluation  *(nat., reg., mun.)* | 1. Data on [waste collection](#waste_collection) and disposal (quantity, type, etc.) is missing [4]. | 1. Waste collection and disposal records are introduced. | 1. Data for all disposed wastes are documented (G.3.3.1) and updated annually. 2. Municipalities and regions are obliged to record waste data and forward it to superior authorities. 3. **Waste data is documented centrally and uniformly.** 4. Different registration and notification procedures (G.8.3.4) are established for various wastes [14] also for its [export](#waste_export) [4] | 1. Registration and notification procedures for different types of waste are standardized [14]. | 1. WM data and other environmentally relevant data are available to the public at all political levels [4]. |
|  |  | 1. Simple management information systems (MIS) [11] to identify, record and analyze waste relevant data e.g. in public administration, are introduced. | 1. Increase use of MIS in public administration. | 1. Innovative MIS (with data warehouses and big data applications) are used to analyze large data amounts user-friendly and more quickly. | 1. A smart WM system is emerging in which waste data is integrated into large data centers and is available in real time [24]. |
|  | 1. Representative waste statistics at national, regional or municipal level are lacking. | 1. Municipal or regional waste statistics (collection and disposal) are created without being publicly available. | 1. Waste statistics are publicly available. 2. Waste balances and forecasts through improved data. | 1. Data basis in the [CE](#CE) enables precise forecasts, meaningful reporting and is a basis for political decisions. | 1. (Supra-)National standardized benchmark indicators to evaluate WM systems exist. |
|  |  |  | 1. **Indicators are used to evaluate** [**WM**](#WM) **systems.** | 1. Use of different indicators for WM systems evaluation and monitoring on all political levels .^[[19]](#footnote-20)^ |  |
|  | 1. Specific [collection](#Collection_rate), disposal, recovery and [recycling rates](#Recycling_Rates) are lacking. | 1. Collection, disposal, recovery and [recycling rates](#Recycling_Rates) are estimated on all political levels. | 1. **Uniform collection, disposal and recycling rates** exist on all political levels. |  | |

|  | **Stage 1**  **Absence or lack of essential elements of WM** | **Stage 2**  **Reliable collection and improved landfill sites** | **Stage 3**  **Separate collection and sorting** | **Stage 4**  **Expansion of the recycling industry** | **Stage 5**  **Circular economy - waste as a resource** |
| --- | --- | --- | --- | --- | --- |
| **Governance – Education and research (G.12 – G.14)** | | | | | |
| **G.12**  Education  *(nat., reg., mun.)*  *Impact on SDG 4.7 and SDG 12.8* | 1. Lack of training opportunities to qualify personnel in [WM](#WM). | 1. **Education and training opportunities** are available to train personnel in the field of WM. 2. Specific job profiles (skilled workers, technicians, engineers, etc.) emerge for urban cleaning, [waste collection](#waste_collection), sorting and disposal. | 1. Emerging of job profiles for [recycling](#recycling) or recovery. 2. WM issues are addressed in professions for which the handling of waste is relevant [24]. | 1. Qualification offers for specialized personnel in the field of [CE](#CE) are expanded and adapted to current developments ([Zero-Waste](#zerowaste), [waste prevention](#waste_prevention), climate protection, etc.). | 1. CE is an integral part of educational courses either for academics, pupils and workers. |
|  |  | 1. Study programs without WM focus are available to train academic specialists (e.g., engineers), who can be employed in WM. | 1. **Environmental science and WM study programs** to train academic staff exist. |  |  |
|  | 1. Lack of educational programs for pupils in schools regarding WM. | 1. WM relevant topics are voluntarily addressed in preliminary and secondary schools. 2. Educational pilot projects are launched selectively, which are financed externally, e.g., by international active donors, NGOs or companies. | 1. **In schools, environmental and waste topics are taught** increasingly on a voluntary basis [29]. | 1. In elementary and secondary schools, environmentally and [CE](#CE) relevant topics (e.g., [recycling](#recycling) and [waste prevention](#waste_prevention)) are included in the curricula to raise awareness among children, adolescents and young adults [30]. |  |
| **G.13**  Research  *(nat., reg.)*  *Impact on SDG 9.5 and SDG 9.5.a* | 1. Lack of faculties or institutes^^[[20]](#footnote-21)^^, studying [WM](#WM) issues on their own or in cooperation with other countries. 2. WM issues are only investigated by foreign disciplines, research institutions or firms. | 1. **Research and development (R&D) faculties** are established at national or regional level [1] to study WM issues. 2. Cooperation’s with foreign research institutions or firms and countries^[[21]](#footnote-22)^ [31] regarding research and education exist. | 1. At national and regional level, the expansion of R&D facilities [1] is promoted intensively. 2. Increasing number of faculties/institutes at national level studying WM issues or having cooperation’s with other countries regarding research and education. | 1. Interdisciplinary research centers are established to study relevant [CE](#CE) issues. |  |
|  | 1. Research activities focus mainly on the setup of [waste collection](#waste_collection) and disposal infrastructures. | 1. Research activities focus mainly on the efficient collection, disposal and treatment of waste, and the minimization of social and economic risks. | 1. Research activities are mainly focused on optimized and efficient [waste recycling](#waste_recycling), risk minimization (social, economic and environmental) and resource efficiency. 2. **WM issues are more likely studied interdisciplinary**. | 1. Research activities focus mainly on the optimization of process flows in the CE (e.g., through digitalization or industrial symbiosis), bioeconomic approaches, waste avoidance and Water-Waste-Soil Nexus [32]. 2. Increase studies exploring [CE](#CE) potentials in companies. | 1. CE related research projects are usually interdisciplinary and holistic. |
| **G.14**  Awareness building  *(nat., reg., mun.)*  *Impact on SDG 12.8* | 1. Lack of awareness campaigns regarding [WM](#WM). | 1. **WM awareness campaigns^[[22]](#footnote-23)^ [4] are carried out on some political levels.** | 1. National and municipal initiatives/projects to raise WM awareness are implemented. | 1. Awareness campaigns especially to promote [waste prevention](#waste_prevention) and closing loops are carried out on all political levels. |  |
|  | 1. Population neglects the importance of WM and lives mainly according to the "not-in-my-backyard" principle [33]. | 1. Awareness regarding knowledge and concerns about WM is increasing in small parts of the population [33]. | 1. Society and individual companies gain experience and acquire a basic understanding of aspects of WM, including in the context of sustainability [33]. | 1. Survey results show positive changes in the attitude regarding waste and [CE](#CE) in large parts of the population and companies. 2. Learned values, interests and the motivation of individuals are reflected in the growing public participation in political decision-making processes (G.9.3.2) regarding CE [33]. | 1. Behavior changing of population and companies enable a sustainable CE [24,33]. 2. Increasing environmental awareness among the population enables the achievement of ambitious CE or [zero-waste](#zerowaste) goals [34]. |
| **Governance – Occupational safety, health and environmental protection (G.15 – G.16)** | | | | | |
| **G.15**  Occupational health and safety  *(reg., mun., comp.)*  *Impact on SDG 3.3, 3.8 and 8.8* | 1. Occupational health and safety measures (OHSM)^[[23]](#footnote-24)^ in [WM](#WM) sector are fully neglected. 2. (Fatal) accidents, illnesses, etc. of personnel [18] occur frequently. | 1. **OHSM in WM [4,16,35,36] are increasingly** implemented in the formal sector and rarely in the [informal sector](#InformalSector). 2. Decrease of work-related accidents, illnesses, etc. are registered firstly in the formal sector. | 1. OHSM (e.g., increased automation) are implemented entirely in formal and increasingly in the informal sector. 2. Decrease of work-related accidents, illnesses, etc. are registered in both sectors. | 1. Due to existing control mechanisms (G.8.3.4 and SM.6.3.7), a high level of occupational health and safety exists. | 1. The number of work-related accidents, illnesses, etc. is reduced to a minimum. |
|  | 1. [Waste picker](#waste_picker)s work and live under poor conditions at waste dumps / landfills [18,21] 2. Children work as waste pickers. | 1. Only adults work at [controlled landfills](#controlled_landfill). 2. Living in dumps or [landfills](#landfill) and child labor is generally prohibited, but still exists. | 1. The prohibition of living on landfills and child labor is fully realized. |  |  |
| **G.16**  Environmental protection  *(nat., reg., mun., comp.)* | 1. Environmental and climate aspects are neglected in decision-making processes in politics, companies and society [21]. | 1. **Environmental and climate protection measures** are integrated in WM. | 1. WM and pollution control are more significant in environmental politics. | 1. WM issues (waste-to-energy, secondary raw materials), are increasingly integrated in resource and energy efficiency concepts [4]. | 1. [CE](#CE) makes a significant contribution to a resource-efficient and low-GHG economy. |
|  |  | 1. [Emissions trading](#EmissionTrading) (G.2.3.4) is applied; but, GHG emissions from WM increase compared to 1990. | 1. Initial emission savings in WM (WD.2.3.2); GHG emissions decrease by ≤ 10% compared to 1990. | 1. GHG emissions related to [CE](#CE) are >10% to ≤ 50% lower compared to 1990. | 1. GHG emissions from CE are > 50% lower compared to 1990. |

|  | **Stage 1**  **Absence or lack of essential elements of WM** | **Stage 2**  **Reliable collection and improved landfill sites** | **Stage 3**  **Separate collection and sorting** | **Stage 4**  **Expansion of the recycling industry** | **Stage 5**  **Circular economy - waste as a resource** |
| --- | --- | --- | --- | --- | --- |
| **Sector and Market (SM.1 – SM.5)** | | | | | |
| **SM.1**  Sector development  *(nat., reg., mun., comp.)* | 1. The existing [WM](#WM) sector is unstructured. | 1. **Institutional structuring of WM** (G.4.2.1, G.5.2.1, G.5.3.1, G.6.2.2 and G.7.2.1) enables creation of a WM sector [18]. | 1. WM is evolving into a relevant economic sector (classification E according to ISIC), so that it is now part of the industrial policy [21]. | 1. The WM sector is growing, especially in the area of [recycling](#recycling) and resource recovery [37]. | 1. Due to close cooperation with other sectors, a functioning CE is implemented that is improving constantly. |
|  | 1. Interests of companies or persons from the WM sector are neglected at the political level due to the lack of a lobby. | 1. Formation of associations and interests groups representing interests of the WM sector. | 1. **Integrative approaches to address WM challenges are developed through increased** **dialogue among various** [**stakeholder**](#stakeholder)**s.** | 1. Initial efforts to collaborate with other sectors to address the challenges of the [CE](#CE) are evolving. |  |
| **SM.2**  Jobs  *(nat., reg., mun., comp.)* | 1. Jobs in the formal sector are available without requiring qualifications for the work performed. 2. Lack of qualified staff and expertise in institutions dealing with [WM](#WM) issues [19,24] | 1. Certain jobs in WM require appropriate qualification, but there is still a lack of qualified personnel [24]. 2. [Waste collection](#waste_collection) and sorting hold great employment potential [21]. | 1. Many jobs are created in WM (especially in the [recycling](#recycling) sector) [21]. 2. Great demand of qualified personnel, especially for the construction and operation of plants. | 1. Additional jobs are created as the recycling industry expands. 2. Increasing recruitment of personnel for in-house [waste disposal](#waste_disposal) and recycling in companies. | 1. Qualified specialized personnel for the development, planning, construction and operation of advanced high-tech facilities are increasingly in demand. |
| **SM.3**  [Informal sector](#InformalSector)  *(nat., reg., mun.)*  *Impact on SDG 8.3* | 1. The informal sector is unstructured and informal workers are self-reliant. | 1. Some informal workers form small communities. | 1. Reorganization of the sector through responsible cooperatives or umbrella organizations [4,18]. | 1. Complete formalization of the informal sector by their integration into formal municipal, private sector enterprises or cooperatives is achieved [23,38]. |  |
|  | 1. Their role and contribution to [WM](#WM) is neglected by policy makers and public authorities [18]. 2. The distrust of informal workers towards the public sector is high. 3. The informal sector is disadvantaged compared to the formal sector^[[24]](#footnote-25)^. | 1. Their role and WM contribution are perceived by policy makers and public authorities. 2. **Start of institutional attempts^^[[25]](#footnote-26)^^ to integrate informal workers [17].** 3. They get support from development banks and NGO’s to improve their rights and working conditions [40]. | 1. Profession “[waste picker](#waste_picker)” is recognized by policy makers and public authorities [39]. 2. Emerging initiatives to integrate them into the formal sector (e.g. [waste collection](#waste_collection) and [recycling](#recycling)) [38,40]. 3. Experiences of waste pickers are actively used to optimize WM activities [41]. |  |  |
|  | 1. Informal sector is accepted by a minority of the society [40]. |  | 1. Informal sector is accepted by a majority of the society. |  |  |
|  | 1. Share of informal workers compared to the total number of employees in WM ≥ 35%. | 1. Share of informal workers compared to the total number of employees in WM < 35% and > 10% [42]. | 1. Share of informal workers compared to the total number of WM employees ≤ 10% [42]. |  |  |
| **SM.4**  [WM](#WM) system structure and organization  *(nat., reg., mun., comp.)* | 1. [Waste disposal](#waste_disposal) is carried out uncoordinated and inefficient by public or private companies and [waste picker](#waste_picker)s. | 1. WM coordination between various actors is improving, but economic, personnel, technical and organizational inefficiencies remain [24]. | 1. [PPP](#PPP)-projects are emerging in the waste sector [4,43]. 2. Companies specialized in [waste recycling](#waste_recycling)/disposal need a specific certification (G.8.3.3) to work in WM. 3. Strict controls (G.8.3.4) increase the bureaucratic burden on WM companies [44]. | 1. Dynamic interplay between small innovative companies and internationally operating large companies in the WM industry exist. |  |
|  |  |  | 1. **A** [**dual WMS**](#DualWM) **(G.2.3.5) or other systems exist** that fulfill disposal and [recycling](#recycling) obligations of manufacturers, retailers and distributors of products (e.g., [packaging](#packaging)). | 1. Dual WMS or other systems are a reliable and efficient waste disposal systems with a large number of service providers. | 1. Dual WMS or other systems ensure that recycling and return rates, reach high levels in order to close the cycles in the best possible way. |
| **SM.5**  Financial funding  *(nat., reg., mun.)* | 1. [WM](#WM) projects are mostly financed externally (e.g., by internationally operating governmental organizations, development banks, NGO’s). | 1. **Higher financial support of national funders for WM development** [28]. 2. Financially weak municipalities receive loans from banks through state guarantees [18]. | 1. Financial support from (inter-)national donors to develop a sustainable WM and [CE](#CE) (incl. energy and resource efficiency) is increasing [45]. 2. Financial supporting of companies to implement clean production measures^[[26]](#footnote-27)^ [9,28] is available. | 1. Financial support from (inter-)national donors to promote CE is increasing, with greater consideration of digitization, [bioeconomy](#BE) and [waste prevention](#waste_prevention). 2. Funding for industrial [28] and urban [46] symbiosis and cooperation between companies and municipalities [28]. | 1. Financial support of R&D projects helps to achieve ambitious CE targets (G.1.5.1). |
|  | 1. Approved funding is inefficiently used or misused, so that many projects fail. 2. Failing subsidies, misfunding and double funding (e.g., due to poor arrangements within public institutions) happen often. | 1. Investments by municipalities are controlled and managed by the state [18]. 2. Conditions for obtaining subsidies are becoming stricter; nevertheless, misuse and inefficient use of approved grants are still prevalent. | 1. **Control mechanisms reduce misuse and inefficient use of funds.** 2. Double funding, failing subsidies, and misfunding are identified^[[27]](#footnote-28)^ and countered. |  | 1. Receiving of (non-)governmental financial support is generally linked to compliance with sustainability criteria. |

|  | **Stage 1**  **Absence or lack of essential elements of WM** | | **Stage 2**  **Reliable collection and improved landfill sites** | **Stage 3**  **Separate collection and sorting** | **Stage 4**  **Expansion of the recycling industry** | **Stage 5**  **Circular economy - waste as a resource** |
| --- | --- | --- | --- | --- | --- | --- |
| **Sector and Market II (SM.6 – SM.7)** | | | | | | |
| **SM.6**  Enterprises  *(comp.)*  *Impact on SDG 9.4 and 12.6* | 1. Companies (manufacturers, distributors, or retailers) neglect [WM](#WM) issues. |  | | 1. Manufacturers, distributors, or retailers of certain products^[[28]](#footnote-29)^ must participate financially or organizationally in WM (**G.2.3.5**) [11]. 2. Companies purchase insurance against environmental damages caused and the insurance industry enforces solutions for potential risks from such damages. | 1. [Zero-Waste](#zerowaste) targets are defined at company level [2]. | 1. Company responsibility and transparency are generally high. |
|  |  |  |  | 1. Companies are starting to design their supply chains in a [circular supply chain](#CSC) manner [12]. | 1. Companies use innovative MIS^^[[29]](#footnote-30)^^ and blockchain to get rapid and user-friendly analysis of large data amounts to improve their processes sustainably [12]. |  |
|  |  |  |  | 1. First company cooperation’s form symbioses regarding their material flows [12]. | 1. Large-scale projects on industrial or urban symbiosis (G.13.4.2 and SM.5.4.2) are implemented (e.g., eco-industrial parks) . |  |
|  | 1. The provision of product information for customers is lacking. |  | | 1. Initially, manufacturers are required to **provide product information** to their customers regarding its composition (e.g. hazardous ingredients) and handling during the use and disposal phases. | 1. Companies are increasingly recording data on material and waste flows, including the disposition or losses to the environment, and provide this information to consumers and WM companies [6,7]. | 1. Products generally contain environmentally relevant information (e.g. Carbon or water footprints, material usage). |
|  | 1. Business reports, containing WM data and measures to improve WM are missing. |  | | 1. First **environmental** **business reports** are published, containing WM data and measures to improve WM. 2. Audits^[[30]](#footnote-31)^ (G.8.3.1) are carried out in companies [28]. 3. Few companies use eco labels and environmental certificates to promote their products. | 1. Environmental or sustainability reports (with inclusion of SDGs) are increasingly published by companies. 2. Eco-Labels are used in mostly all products to inform the consumers regarding environmental impact, use of biobased materials, recycled materials, etc. | 1. Environmental and sustainability reports are published by domestic and foreign companies with which trade is conducted [9]. |
| **SM.7**  Recycling market  *(nat., reg., mun.)* | 1. Markets for waste and recyclables are small, mostly informal and unregulated; trade structures are non-transparent. | | 1. Recycling markets are expanding and are starting to self-regulate, but trading structures remain non-transparent. | 1. **Trading structures for waste and recyclables**^[[31]](#footnote-32)^ are regulated and transparent [4,21]. 2. Price fluctuations of certain secondary materials and fuels are regulated by governmental measures. 3. Exchange platforms for secondary products and wastes emerge. | 1. Strengthening of the secondary resource market through government incentives (G.2.4.1) [6,21]. 2. Strong connection of companies regarding exchange and trade of secondary materials, by-products or other materials for reuse [21,47]. 3. New actors (e.g. volunteers) participate in [collection of recyclables](#recyclables_collection); revenues are invested in social or environmental projects. | 1. Automated logistics and market platforms allow simplified searching and purchasing of waste or secondary materials [37]. |
|  |  |  |  | 1. Secondary materials provide a small portion of industrial demand and generate revenues [21]. | 1. Secondary materials cover a large part of the industrial demand for raw materials [21] | 1. Supply risks are reduced through a systematic [CE](#CE). |
|  |  |  |  | 1. It is difficult for high-quality recyclates to enter the market or its use is inadequate (firing instead of [recycling](#recycling)) [37]. 2. Secondary resources are perceived to be of low value due to missing quality information [37]. | 1. Pure plastic recyclates volumes increase (G.1.4.2, G.2.4.1, CT.4.3.1 and WR.4.4.2), while the volume of mixed plastics recovered decreases. 2. Improved information on secondary materials quality reduces biases. | 1. Strict limits (G.10.4.2) enable high quality recyclates. |
|  | 1. Import and [export](#waste_export) of waste (including electronic waste, shipwreck, etc.) and recyclables, is unregulated and occurs without compliance with any requirements. | | 1. Trading of wastes and recyclables occurs predominantly in the country. 2. Restrictions may be imposed for import and export of wastes and recyclables (G.10.2.2), but controls occur selectively. | 1. International trade with other countries regarding waste and recyclables is increasing. 2. Initial import/export bans on [hazardous waste](#hazardous_waste)s exist (G.2.3.4), but insufficient control encourages illegal transport. | 1. **Introduction of stricter import and export criteria** for (non-)hazardous, hardly recyclable wastes and used goods^[[32]](#footnote-33)^ [6]. | 1. Smart [WM](#WM) system enables real-time networking [24], so that secondary resources^[[33]](#footnote-34)^ can automatically be traded globally [37]. |

|  | **Stage 1**  **Absence or lack of essential elements of WM** | **Stage 2**  **Reliable collection and improved landfill sites** | **Stage 3**  **Separate collection and sorting** | **Stage 4**  **Expansion of the recycling industry** | **Stage 5**  **Circular economy - waste as a resource** |
| --- | --- | --- | --- | --- | --- |
| **Collection and transport (CT1. – CT.8)** | | | | | |
| **CT.1**  [Waste collection](#waste_collection)  *(mun.)* | 1. Lack of regulated and systematic collection [4], collection is limited to important areas^[[34]](#footnote-35)^. 2. Uniform waste bins for collection are missing. | 1. A systematic, reliable and regulated collection [4,21] is ensured, but carried out in an inefficient manner (economically, organizationally) [18]. 2. Uniform and compatible aboveground systems are primarily used in waste collection. 3. Collection points are within 200m distance [48]. | 1. Measures (e.g. waste locks, QR-tracking), concepts to optimize waste collection (collection intervals^[[35]](#footnote-36)^) and route planning make waste collection more efficient. 2. New waste bin systems, such as waste locks, are used often in densely populated areas. 3. Available service for cleaning up bins from households. | 1. Underground systems^[[36]](#footnote-37)^ are installed in public spaces. 2. Waste bins with fill level sensors are used [24,49]. 3. More digitalization^[[37]](#footnote-38)^ measures are introduced to increase collection efficiency. | 1. Waste bins without negative effect on the urban landscape are in public spaces. 2. Avoidance of overfilling by using smart waste bins. 3. Smart waste bins recognize types of waste and separate them automatically [24]. |
|  | 1. Basic principles of urban hygiene are neglected [4,21], major littering exists [48]. | 1. Principles of urban hygiene are partially met [4] by regular urban cleaning and emptying public bins, but littering in public spaces is still a problem [48]. |  |  | 1. Principles of urban hygiene are fully maintained [4] and littering in public spaces is not a problem anymore [48]. |
| **CT.2**  Service provider  *(mun.)* | 1. [Waste collection](#waste_collection) is carried out partially or predominantly by [waste picker](#waste_picker)s [4]. | 1. Waste collection is mainly done by the formal sector; existing collection gaps are filled by waste pickers. | 1. Waste collection is carried out exclusively by the formal sector or by an organized [informal sector](#InformalSector) (SM.3.3.1). |  | 1. Waste collection is carried out exclusively by the formal sector (SM.3.4.1). |
| **CT.3**  [Collection rates](#Collection_rate)  *(mun.)*  *Impact on SDG 11.6* | 1. Collection rate:  - urban: ≤ 50% OR no data - rural: ≤ 30% OR no data | 1. Collection rate:  - urban: > 50% and ≤ 70% - rural: > 30% and ≤ 50% | 1. Collection rate:  - urban: > 70% and ≤ 80% - rural: > 50% and ≤ 80% | 1. Collection rate:  - urban: > 80% and ≤ 95% - rural: > 80% and ≤ 95% | 1. **Collection rate:**  - **urban: > 95% and ≤ 100%** - **rural: > 95% and ≤ 100%** |
| **CT.4**  [Separate collection](#seperate_collection)  *(mun.)* | 1. Residual waste from households and (small) businesses is collected collectively. 2. Solely informal workers collect profitable recyclables directly from households or [landfills](#landfill) [21]. | 1. First forms **of separation at source collection** (e.g. metals, organics, glass or paper, wet/dry, etc.) emerge (e.g. separation in 2 fractions) [48]. 2. Recyclables are collected also by the formal sector. | 1. **Separation at source collection for households and businesses is mandatory**, with few exceptions (G.2.3.1). Separation in 3 or more fractions is implemented [48]. 2. Recycling centers are set up for the collection of various types of waste (incl. [hazardous waste](#hazardous_waste)). | 1. Measures are introduced to reduce the illegal collection of e.g. used electronics [51]. | 1. All recyclables are collected separately (G.2.4.1). |
|  |  | | 1. Separate collection leads to a better recovery of recyclables, but the rate of incorrect sorting is > 30%. | 1. Rate of incorrect sorting of recyclables is between > 10% and ≤ 30%^[[38]](#footnote-39)^. | 1. Rate of incorrect sorting of recyclables is ≤ 10%. |
|  |  | 1. Start of separate collection of hospital waste in healthcare facilities (G.5.2.4). | 1. Official separate collection of electrical, construction and demolition, agricultural and forestry waste (G.5.3.7). |  |  |
| **CT.5**  [Collection of recyclables](#recyclables_collection)  *(mun.)* | 1. Recyclables collection rates are missing, only estimations are available or data is not publicly available. | 1. Recyclables^[[39]](#footnote-40)^ collection rate (G.11.3.3):  - Metals: ≤ 50% - Paper: ≤ 30% - Glass: ≤ 30% - Organics: ≤ 30% - Plastics: ≤ 10% - Textiles: ≤ 10% | 1. Recyclables^39^ collection rate:  - Metals: > 50 and ≤ 70% - Paper: > 30 and ≤ 70% - Glass: > 30 and ≤ 70% - Organics: > 30 and ≤ 70% - Plastics: > 10 and ≤ 50% - Textiles: > 10 and ≤ 50% | 1. Recyclables^39^ collection rate:  - Metals: > 70 and ≤ 90% - Paper: > 70 and ≤ 90% - Glass: > 70 and ≤ 90% - Organics: > 70 and ≤ 90% - Plastics: > 50 and ≤ 90% - Textiles: > 50 and ≤ 90% | 1. Recyclables^39^ collection rate:  - Metals: > 90% - Paper: > 90% - Glass: > 90% - Organics: > 90% - Plastics: >90% - Textiles: >90% |
| **CT.6**  Waste transport  *(mun.)* | 1. Formal sector: Use of simple small vehicles (e.g., small trucks, pick-ups). | 1. Formal sector: Mostly large vehicle fleet and more automation available for [waste collection](#waste_collection); use of large waste collection vehicles. | 1. Formal sector: Waste logistics companies improve vehicle fleets [4]. Waste collection vehicles with compactors and lifters take over waste collection [21]. | 1. Use of different collection vehicles depending on waste^[[40]](#footnote-41)^. | 1. Highly engineered and fully automated collection vehicles facilitate collection through integrated false waste detection, quantity calculation and automatic data collection and transfer. |
|  | 1. [Informal sector](#InformalSector): Only use of pack animals or simple handcart, wheelbarrows or bicycles. | 1. Informal sector: Mostly use of modified wheelbarrows, motorcycles, small vehicles. | 1. Informal sector: Mostly use of modernized small trucks. | See SM.3.4.1. | |
| **CT.7**  [Transfer stations](#TransferStation)  *(mun.)* | 1. Waste is transported directly to [landfills](#landfill) or dumps by waste collectors. 2. Road sections are used as transfer areas. | 1. **Decentralized transfer stations** at favorable traffic nodes are introduced [21]. 2. Transfer stations are simple and unrestricted accessible for public. | 1. Transfer stations increase collection efficiency. 2. Transfer stations are advanced (use of differentiated collection, handling and storage techniques [4]) and with restricted access for public. |  | 1. All existing decentralized transfer stations correspond to the current state of the art. |
| **CT.8**  Collection of [hazardous wastes](#hazardous_waste)  *(mun.)*  *Impact on SDG 6.3 and 12.4* | 1. Hazardous waste from households and (small) businesses are collected together with other waste fractions [4]. | 1. Small quantities of hazardous waste from households are collected separately through designated public waste containers. | 1. Small quantities [4] can be disposed free of charge. | 1. All hazardous waste flows are collected separately (G.2.4.1). | 1. Hazardous waste quantities from households and industry are reduced to a minimum (PR.4.4.2 and PR.4.5.2). |
|  | 1. Lack of [separate collection](#seperate_collection) of hazardous waste from industry. |  | 1. **Hazardous waste from industry is collected almost completely separated** [4]. |  |  |

|  | **Stage 1**  **Absence or lack of essential elements of WM** | **Stage 2**  **Reliable collection and improved landfill sites** | **Stage 3**  **Separate collection and sorting** | **Stage 4**  **Expansion of the recycling industry** | **Stage 5**  **Circular economy - waste as a resource** |
| --- | --- | --- | --- | --- | --- |
| **Waste disposal (WD.1 – WD.5)** | | | | | |
| **WD.1**  [Waste disposal](#waste_disposal)  *(nat., reg., mun.)*  *Impact on SDG 6.3, 11.6, 11.b* | 1. Lack of targeted and centralized waste disposal [4]. 2. Wastes (incl. [hazardous wastes](#hazardous_waste)) are mainly disposed in [open dumps](#open_dump), uncontrolled landfills, roads, open areas and in water (river, sea) [19]. 3. Waste is openly burned for better recovery of metals or used as fuel in households for heating and cooking [21]. | 1. Targeted and centralized waste disposal in minimum [controlled landfills](#controlled_Landfill) [4,21] is regulated by law (G.2.2.1). 2. [Open burning](#open_burning) and [waste dumping](#waste_dumping) are illegal, but still practiced due to lack of controls (G.8.2.3), collection gaps or other inefficiencies. | 1. [Landfill](#landfill) classes^[[41]](#footnote-42)^ with differentiated disposal requirements for various wastes are introduced [4]. 2. **Hazardous and non-hazardous waste are no longer disposed mixed on landfills**. 3. Liquid waste are no longer be disposed in landfills. 4. (In)active landfill forms with low standards are converted to [sanitary landfills](#sanitary_landfill) [1]. | 1. Predominantly mineral waste is landfilled [4]. 2. Recyclables are rarely landfilled [45,53]. 3. The after-use of former landfill sites after their closure is becoming more relevant. 4. Waste is disposed exclusively on sanitary landfills. 5. Approved sites allow the municipality to quickly and safely dispose excessive waste in disaster [20]. | 1. Waste landfilling is becoming less important. 2. Very small waste quantities are landfilled [4]. 3. Closed landfill areas are usually reused for other purposes, so that they are available safely. 4. The rehabilitation of old landfills with potential [recycling](#recycling) options is gaining in importance [4]. 5. Illegal dumping is fully avoided. |
|  | 1. This [landfill](#landfill) status predominates: [open dump](#open_dump) or [controlled dump](#Controlled_dump) | 1. This landfill status of active landfills predominates: [controlled landfill](#controlled_landfill) or [engineered landfill](#Engineered_landfill) |  | 1. This landfill status of active landfills predominates: **sanitary landfill**. | 1. All existing (in)active landfills have been almost converted to sanitary landfills. |
| **WD.2**  Operational measures  *(reg., mun.)* | 1. Lack of disposal controls such as waste registration (type, quantity or origin). 2. Waste is burned on dumping sites [48]. 3. Landfill access controls are lacking or inadequate [48]. 4. Dumping sites are not staffed or only in short times for site checking [48]. | 1. Registration of waste type, quantity and origin and weighing of waste before disposal. 2. Waste burning on landfill sites is missing [48]. 3. Placement and compaction of waste with daily use of cover material is mainly implemented [48,54]. 4. Site drawing showing landfill boundary and filling area in place [48]. 5. Site is staffed during operational hours [48]. | 1. Registration and compaction of waste are the norm. 2. **A systematic pre-treatment of waste is mandatory (G.2.3.1) [4].** 3. A systematic landfill management is carried out for the placement of the landfilled waste. 4. Measures for final covering, closure and post-closure management are realized [54]. |  | 1. All existing and new landfills comply with the current state of the art, so that they pose little to no risk to human health or the environment. |
| **WD.3**  [Leachate](#LFL) water management  *(reg., mun.)*  *Impact on SDG 6.3* | 1. Leachate infiltrates the soil or evaporates. 2. Surface and groundwater monitoring is lacking [54]. | 1. Leachate collection systems are available [54]. 2. Recirculation of leachate to the landfill body is often implemented as treatment option. 3. Surface and groundwater monitoring is implemented [54]. | 1. Leachate collection, treatment and monitoring is established and carried out in all active landfills. | 1. Strict limits (G.10.3.2) are fulfilled for surface and groundwater monitoring [54]. | 1. Appropriate measures lead to a high decrease of leachate and a reduction in its toxicity, so that it no longer poses any health or environmental risks. |
| **WD.4**  [Landfill gas](#LFG) management  *(reg., mun.)* | 1. Landfill gas is released into the atmosphere [54]. | 1. Passive gas collection or flaring of landfill gas is implemented as a measure. | 1. Active landfill gas collection for further [energy recovery](#energy_recovery) and gas monitoring takes place [54]. | 1. Strict limits (G.10.3.2) are fulfilled for landfill gas monitoring. | 1. Controlled flaring of landfill gas is done only in case of emergency ("emergency flaring") and when it is unprofitable (e.g., at small gas quantities). |
| **WD.5**  Other means of disposal  *(nat., reg., mun.)* | 1. Waste burning or dumping in high seas and in coastal waters takes place. |  | 1. By joining international agreements (London, Bamako) (G.2.3.4) waste burning or dumping in high seas and in coastal waters is prohibited. But, they still take place due to lack of controls. |  | 1. Strict and regular controls (G.8.4.4) and greater environmental awareness (G.14.4.2) lead to the avoidance of waste burning or dumping in high seas and in coastal waters. |

|  | **Stage 1**  **Absence or lack of essential elements of WM** | **Stage 2**  **Reliable collection and improved landfill sites** | **Stage 3**  **Separate collection and sorting** | **Stage 4**  **Expansion of the recycling industry** | **Stage 5**  **Circular economy - waste as a resource** |
| --- | --- | --- | --- | --- | --- |
| **Energy recovery (ER.1 – ER.3)*** | | | | | |
| **ER.1**  Thermal disposal and [energy recovery](#energy_recovery)  *(reg., mun.)* | 1. A controlled energy recovery or thermal disposal of waste is missing. | Pre-check before implementing an incineration plant**:  To successfully implement waste [incineration](#incineration) plants, these organizational criteria [55–57] must be respected:   - *Waste is disposed in* [*controlled landfills*](#controlled_landfill) *(WD.1.2.3).* - *Qualified staff is available (G.12.2.1 and G.12.2.3).* - *Collection is systematic and organized (CT.1.2.1).* - *Sorting at source is implemented to control waste, which is not suitable for incineration (CT.4.2.1).* - *Basic laws and rules on WM have been developed (G.2.2.1 and G.2.3.1).* - *Incineration plants are positioned in upper level plans (master plans, national strategies, etc.) (G.4.2.4 and G.5.2.3).* - *Political and social will for the installation is given.* - *Total costs for the plant are secured.* - *Public or private actors shall be experienced in managing incineration plants efficiently.* - *Most spare parts for maintenance can be purchased locally. Sales offices for spare parts to be imported are locally available.*   *These technical criteria [55–57] are essential:*   - *Supply of combustible waste must be stable over the year and at least 50,000 Mg/year.* - *Lower heating value must be at least 7 MJ/kg on average and must always be above 6 MJ/kg in any season.* - *Bottom ash and fly ash can be treated safely.* | 1. Energy recovery or thermal disposal is increasingly used to treat large quantities of the cities’ waste (e.g., residual, bulky, forestry, hazardous and industrial waste) inside or outside the country. 2. Depending on the organizational affiliation of the installation, irrevocable agreements are in place that regulate waste delivery, energy sales, and pricing [55]. 3. At least 150.000 tons per year of suitable waste fractions are available for incineration [57]. | 1. Although material [recycling](#recycling) increase, energy recovery continues to be an essential treatment alternative. 2. The lower heating value is at least 11 MJ/kg on average. | 1. The amount of waste for energy recovery tends to decrease as waste is recycled more (WR.7.5.1). |
|  | 1. Co-incineration of waste is missing. | 1. Specific wastes or [RDFs](#RDF) are co-incinerated in industrial firing plants (e.g., cement plants) in compliance with specific quality standards (G.10.2.2). | 1. Energy recovery establishes as an alternative to landfilling; energy-intensive industries (blast furnaces, cement and power plants) often make use of it [4]. |  |  |
| **ER.2**  [Incineration](#incineration) plants  *(reg., mun.)* | 1. Waste incinerators are neither in operation nor planned in the country. | 1. Incineration plants^[[42]](#footnote-43)^ (incl. flue gas cleaning - FGC) for controlled [energy recovery](#energy_recovery) or thermal disposal can be in planning or construction stage in the country or simple operating incineration plants, which are engineered with process controls, but without an FGC can be assigned to this stage. At least operating parameters (temperature, smoke etc.) shall continuously be monitored and recorded [48]. | 1. Only waste incinerators with FGC are in use^^[[43]](#footnote-44)^^, which meet this requirements [48]:  - Continuous monitoring of operating parameters and emissions. - Monitoring systems are routinely calibrated. - Maintenance plans or schedules are in place. - Evidence that equipment is well maintained. - Fire extinguishers available on site. | 1. High-tech and state of the art processes and equipment are used to ensure high operational reliability and safe destruction of organic pollutants and a safe removal of heavy metals and other compounds [7]. 2. Flue gas controls compliant with applicable environmental standards (G.10.4.1) [48]. | 1. Existing data concerning composition and quality of the incinerated waste in combination with the integration of digital measures ensures an automatic and homogeneous waste input. |
| **ER.3**  Energy and raw material recovery  *(reg., mun.)*  *Impact on SDG 7.1 and 7.2* | 1. Raw material production and [energy recovery](#energy_recovery) through controlled waste [incineration](#incineration) is missing. |  | 1. Recovered energy from incineration is sold as electrical or thermal energy (e.g. district heating or steam for industrial purposes) or as a combination of both. [48,55] 2. Incineration or RDF plants are located in an area with moderate heat demand. Good transport and energy infrastructure exists [57]. |  | 1. Incinerators and other thermal [recycling](#recycling) facilities are increasingly replacing primary energy sources [21]. |
|  |  |  | 1. Recovered metals from the incineration process are sold; other by-products (gypsum, salts, slags, bottom and fly ash etc.) are treated safely, but recycled rarely. | 1. Slags are increasingly recycled and reused for example in road construction in compliance with limit values (G.10.4.2). | 1. All by-products from waste incineration are almost completely reintroduced into the economic cycle due to high quality standards (G.10.5.1) and established policy incentives (G.2.4.1). |

***Note: Incineration plants are a possible, but not a mandatory treatment option. In case incineration plants are planned or in operation, the component energy recovery should be considered in the assessment.**

****Note: This section is exceptionally structured differently. Here, the preconditions for the construction of a waste incineration plant are presented. These criteria are only to be considered if there is no incineration plant in the case study, yet. If one plant exists, then this section can be neglected.**

|  | **Stage 1**  **Absence or lack of essential elements of WM** | **Stage 2**  **Reliable collection and improved landfill sites** | **Stage 3**  **Separate collection and sorting** | **Stage 4**  **Expansion of the recycling industry** | **Stage 5**  **Circular economy - waste as a resource** |
| --- | --- | --- | --- | --- | --- |
| **Waste recycling (WR.1 – WR.8)** | | | | | |
| **WR.1**  [Waste recycling](#waste_recycling)  *(reg., mun.)* | 1. Material [recycling](#recycling) is rarely done, but [open-loop recycling](#open_loop_RE) and [downcycling](#downcycling) predominate [58]. |  | 1. [**Waste treatment**](#waste_treatment) **is extended by including mechanical, biological, and chemical-physical treatment components [4].** | 1. (Semi-)[closed-loop recycling](#closed_loop_RE) and [upcycling](#upcycling) increase [58]. 2. Dialogues between manufactures and recyclers and other relevant [stakeholder](#stakeholder)s take place to identify and solve recycling problems jointly [6]. | 1. (Semi-) closed-loop recycling dominates [7]. 2. A circular [bioeconomy](#BE) is implemented where technically possible [6]. |
|  | 1. Ensured input for recycling plants is lacking. |  | 1. Input for recycling plants is partly ensured. | 1. Input for recycling plants is fully ensured (G.2.4.1). |  |
| **WR.2**  [Composting](#composting)  *(reg., mun.)* | 1. [Organic waste](#organic_waste) is landfilled or recycled through home composting or used to feed animals. | 1. The majority of organic waste from households, businesses, from parks and markets are recycled in simple open or covered composting facilities or with the help of mobile aggregates [21]. | 1. Introduction of static, closed composting in tunnels and containers including sorting technologies to reduce interfering materials in compost (G.10.3.3). | 1. Dynamic or quasi-dynamic processes are integrated in existing composting plants. | 1. Microplastics can be removed from compost to a very large extent [6]. |
|  |  | 1. Low compost quality and small use in agriculture. | 1. The use of compost in agriculture and landscape management is increasing due to better qualities. | 1. Compost is of very high quality (G.10.4.2), but still contains pollutants such as microplastics. 2. Use of compost in the private sector is increasing. | 1. Compost has a very high quality and purity level. |
| **WR.3**  Fermentation  *(reg., mun.)* | 1. Fermentation plants are neither in operation nor planned. | 1. Fermentation plants may be in the planning stage or under construction. | 1. Fermentation plants are in operation to recycle organics from households, agriculture and forestry. |  | 1. Biogas plants as biorefineries are an essential component for [CE](#CE), [bioeconomy](#BE) and energy supply. |
|  |  |  | 1. Separation of impurities, such as water and H2S, in order to utilize biogas mixtures energetically (e.g. in combined heat and power plants). | 1. Introduction of a CO_2_ separation stage at plants with a min. capacity of 30,000 Mg/a to get natural gas quality. 2. Biomethane is fed into the natural gas grid or used as fuel in combustion engines. |  |
|  |  |  | 1. Nutrient-rich fermentation residues are used as fertilizers in agriculture (G.10.3.3). | 1. Digestates are of very high quality (G.10.4.2), but still contain pollutants such as microplastics. | 1. Mostly full removal of microplastics from digestates by new treatment techniques. |
| **WR.4**  Sorting and recycling plants  *(reg., mun.)*  *Impact on SDG 12.4* | 1. Sorting of recyclables (e.g. metals, plastics, paper, glass, textiles) is done manually. 2. Electrical and electronic waste is rarely or very inefficiently dismantled and without any protective measures. Only valuable materials (metals, etc.) are recovered. | 1. Partially automated operating sorting plants with simple shredding and separation units (e.g., air classifier, magnetic separator) are used for better [recycling](#recycling). 2. Use of mechanical-biological plants for residual waste pre-treatment to recover metals and high-calorific fractions ([RDFs](#RDF)). | 1. Operative sorting plants include mechanical separation stages, screening and visual aggregates to increase the efficiency of hand sorting [21]. 2. Optical separation aggregates allow the production of high quality mono-fractions [21]. 3. Stabilate processes are used for pre-treatment of residual waste to separate metals and [RDFs](#RDF). | 1. Increased use of separation processes for plastics and color sorting (e.g., spectral, X-ray, LIBS) [21,59]. 2. Modern sorting facilities produce high quality individual fractions from separately collected waste [21]. 3. Raw material recycling (e.g., solvolysis, pyrolysis, gasification) of plastic waste is performed to produce e.g., synthesis gases, methanol [60]. | 1. Recycling technologies are increasingly digitalized [24]. 2. Increasing implementation of digitalization measures^[[44]](#footnote-45)^ to rise the recyclability of products. 3. Ensure safe recycling of waste containing harmful substances through improved technical processes for the removal of pollutants [7]. |
| **WR.5**  Refuse derived fuel ([RDFs](#RDF))  *(reg., mun.)* | 1. Lack of RDFs recovery from waste. | 1. Start of recovery of RDFs (high-calorific fractions for [energy recovery](#energy_recovery)) from [waste treatment](#waste_treatment) plants. | 1. Increased recovery of RDFs from waste [4,21]. |  | 1. Amount of RDFs is decreasing due to higher closed loop recycling. |
| **WR.6**  [Recycling](#recycling) of [construction and demolition waste](#CDWaste) (C&D)  *(reg., mun.)* | 1. Only conventional demolition of (non- )residential buildings occurs. | 1. Mostly a conventional demolition of (non- )residential buildings occurs, but semi-selective deconstruction increases. | - 1. Semi-selective demolition occurs predominantly, but selective demolition increases. | 1. Selective demolition predominates. | 1. Only selective demolition occurs. |
|  | 1. Depollution is carried out rarely. | 1. Depollution is carried out more frequently. |  | 1. Depollution is usual practice in demolition. |  |
|  | 1. C&D [waste disposal](#waste_disposal) on [landfills](#landfill) or dumps predominates. | 1. Under certain conditions [61], C&D material is used as filler for surface excavations or landfill construction. | - 1. Quantity of C&D materials as filler for surface excavations and for landfill construction increases.   2. New recycling routes as using C&D material in e.g., road construction, are emerging in compliance with strict limits. | 1. Increase in high quality recycled construction materials (e.g., substitutes for building and road construction) [6]. 2. Heavy or non-recyclable construction materials are treated thermally or landfilled. 3. Robotics are used for efficient sorting of C&D waste [62]. | 1. Heavy or non-recyclable construction materials are no longer allowed for building or road construction. 2. C&D waste is almost exclusively recycled, a marginal quantity is landfilled. |
| **WR.7**  [Recycling rates](#Recycling_Rates)  *(nat., reg., mun.)* | 1. Recycling rates can be high for certain recyclables (e.g., metals), but due to a lack of data, precise numbers are missing. | 1. Recycling rates^39^ of [household waste](#household_waste):  - Metals: ≤ 40 % - Paper: ≤ 40 % - Glass: ≤ 40 % - Composites: ≤ 20 % - Plastics: ≤ 20 %  1. Recycling rates for E-Waste and C&D waste:  - E-Waste: > 10 and ≤ 40 % - C&D waste: > 10 and ≤ 40 % | 1. Recycling rates^39^ of household waste:  - Metals: > 40 and ≤ 70 % - Paper: > 40 and ≤ 70 % - Glass: > 40 and ≤ 70 % - Composites: > 20 and ≤ 60 % - Plastics: > 20 and ≤ 60 %  1. Recycling rates for E-Waste and C&D waste:  - E-Waste: > 40 and ≤ 70 % - C&D waste: > 40 and ≤ 70 % | 1. Recycling rates^39^ of household waste:  - Metals: > 70 and ≤ 90 % - Paper: > 70 and ≤ 90 % - Glass: > 70 and ≤ 90 % - Composites: > 60 and ≤ 90 % - Plastics: > 60 and ≤ 90 %  1. Recycling rates for E-Waste and C&D waste:  - E-Waste: > 70 and ≤ 90 % - C&D waste: > 70 and ≤ 90 % | 1. Recycling rates^39^ of household waste:  - Metals: > 90 % - Paper: > 90 % - Glass: > 90 % - Composites: > 90 % - Plastics: > 90 %  1. Recycling rates for E-Waste and C&D waste:  - E-Waste: > 90 % - C&D waste:> 90 % |
| **WR.8**  [Diversion rate](#DR) (DR) and [Circular material use rate (CMUR)](#CMUR)  *(nat.)*  *Impact on SDG 8.4, 12.2 and 12.5* | 1. DR: ≤ 0,10 DR or no data 2. CMUR: ≤ 1 % CMUR OR no data | 1. DR: > 0,10 and ≤ 0,20 DR 2. CMUR: > 1 % and ≤ 10 % CMUR | 1. DR: > 0,20 and ≤ 0,40 DR 2. CMUR: > 10 % and ≤ 30 % CMUR | 1. DR: > 0,40 and ≤ 0,70 DR 2. CMUR: > 30 % and ≤ 70 % CMUR | 1. DR: > 0,70 and ≤ 1,00 DR 2. CMUR: > 70 % CMUR |

|  | **Stage 1**  **Absence or lack of essential elements of WM** | **Stage 2**  **Reliable collection and improved landfill sites** | **Stage 3**  **Separate collection and sorting** | **Stage 4**  **Expansion of the recycling industry** | **Stage 5**  **Circular economy - waste as a resource** |
| --- | --- | --- | --- | --- | --- |
| **Prevention and reuse (PR.1 – PR.6)** | | | | | |
| **PR.1**  Prevention  *(nat., reg., mun.)*  *Impact on SDG 8.4, 12.2 and 12.4* | 1. [Waste prevention](#waste_prevention) is motivated by poverty. | 1. Waste prevention is predominantly motivated by lack of resources (e.g. materials, metals). |  |  | 1. Decoupling of GDP and [waste generation](#Waste_generation) has been achieved through a functioning [CE](#CE) [4]. |
|  |  |  | 1. Food waste is given a higher political priority. | 1. Initial targets to avoid of food waste are formulated and measures^[[45]](#footnote-46)^ implemented [6]. | 1. Amount of food waste is reduced to a minimum by successful implementation of measures. |
|  |  |  | 1. Few disposables (e.g. plastic bags) is banned or only permitted exceptionally. | 1. All disposables are banned or permitted exceptionally. 2. Prohibited destruction of unsold durable goods (e.g., returns from online sale) [6]. | 1. Increase of substance/product bans^[[46]](#footnote-47)^ (G.2.3.1). 2. [CE](#CE) reduces negative impacts of materials and products by avoiding resources or waste [7]. |
|  |  |  | 1. Initiatives (e.g., 3R) are emerging which, in addition to closing loops, also aim to educate the population. |  | 1. [CE](#CE) provides a long use of raw materials (incl. products, buildings, etc.) in the cycle [7]. |
|  |  |  |  | 1. Lifetime-extension of buildings is ensured before construction phase [6,7]. | 1. The full deconstruction of buildings after use, is ensured, before their realization. |
|  |  |  |  |  | 1. [Waste prevention](#waste_prevention) and [life cycle](#LCA) considerations are the basis of consumption decisions [4,21]. |
| **PR.2**  Circular business and usage models  *(comp.)* | 1. Circular business and usage models are lacking. | 1. Spare parts of end-of-life (EoL) products are used to repair electronic devices or vehicles. | 1. Increase use of spare parts of EoL products to repair electronic devices or vehicles. 2. Repair of products (e.g., electronics, textiles, furniture) is used by a small part of the population. | 1. Repair of products is used by a large part of the population. | - 1. Permanent provision of spare parts through increased digitalization of process and production chains (e.g., with 3D printing) [64]. |
|  |  |  | 1. Minimum online market for spare parts exists. | 1. Various online spare part markets exist. | - 1. Spare part markets are integral part of CE [37]. |
|  |  |  | 1. [Product service systems](#PSS), such as sharing and leasing business models, are offered. |  | - 1. Sharing and leasing models are an integrative part of [CE](#CE) [37]. |
|  |  | 1. Voluntary take-back systems are in place for certain wastes (e.g., pharmaceuticals, batteries). | 1. Introduction of mandatory take-back or [deposit-refund systems](#deposit_refund_system) for waste (e.g. beverage [packaging](#packaging)) [4,29]. | 1. Deposit obligation is extended to all single-use beverage packaging and deposit contribution is higher compared to reusable packaging [6]. 2. Introduction of deposits on other material-relevant products (e.g., smartphones, tablets) or other financial incentives for returning products [51]. | - 1. Innovative take-back and collection systems enable reuse and high-quality [recycling](#recycling) [7]. |
| **PR.3**  Product design  *(comp.)* | 1. Companies neglect end-of-life (EoL) issues of their products. 2. Innovative composite materials are causing disposal problems due to the negligence of EoL in the product design phase. 3. Companies neglect issues such as recyclability, material savings or substitution of hazardous substances in product design phase. | 1. Some companies (pioneers) are considering EoL issues of their products. 2. Some companies consider issues such as recyclability, material savings or substitution of hazardous substances in product design phase. | 1. Through [eco-design](#eco_design), several manufacturers are beginning to design their products in a more environmentally friendly way (e.g. increase recyclability, material savings and substitution of harmful substances) [4,9]. | 1. Increased use of homogenous easy separable, harmless and secondary materials; less use of disruptive substances (= decrease of composites). 2. Recyclability, upgradability and durability is generally considered in product design phase [6]. | 1. High use of secondary, recyclable or biobased materials for product manufacturing [12]. 2. EoL concepts for innovative products prior to market introduction are elaborated to minimize problems at the EoL phase [12,15]. 3. High product recyclability is one basic condition for products entering the market. |
|  |  |  |  | 1. Increased assessment of environmental impacts and resource consumptions of products during their entire [life cycle](#LCA) (cradle-to-grave). | 1. Almost all companies consider all life cycle phases during the product design phase. |
|  |  |  | 1. Voluntarily companies are setting minimum use rates for secondary resources in their product manufacturing. | 1. Legal minimum use rates for recyclates are between ≥30 % and <60 % to produce plastic [packaging](#packaging) and other products [10]. | 1. Legal minimum use rates for recyclates are at least 60 % to produce plastic packaging or other products. |
| **PR.4**  Process optimization and operational disposal strategies  *(comp.)* | 1. Companies neglect issues regarding the proper disposal of their waste and waste avoidance. | 1. Some companies (pioneers) from manufacturing, trade and service sectors are considering how to improve [waste disposal](#waste_disposal) and prevent waste. | 1. Evaluation of production processes to minimize waste and identify [recycling](#recycling) potentials [47]. | 1. Increasing investigation of resource efficiency potentials of production processes (life-cycle perspective) and implementation of measures^[[47]](#footnote-48)^. | 1. Waste avoidance and closing of loops are common practice in medium-sized and large companies. |
|  |  |  | 1. Joining international agreements (G.2.3.4) lead to the substitution of some hazardous materials in manufacturing with less hazardous materials. | 1. Several companies are substituting hazardous substances with non-hazardous substances [47]. | 1. Use of pollutants in [CE](#CE) is possible, if a controlled circulation is ensured in safe, application-based cycles with tolerance thresholds [6,7]. |
| **PR.5**  [Waste generation](#Waste_generation_indicator)^[[48]](#footnote-49)^  *(nat., reg., mun.)*  *Impact on SDG 12.5* | 1. Waste generation per capita and day:  - ≥ 2,0 kg | 1. Waste generation per capita and day:  - ≥ 1,5 and < 2,0 kg | 1. Waste generation per capita and day:  - ≥ 1,0 and < 1,5 kg | 1. Waste generation per capita and day:  - ≥ 0,5 and < 1,0 kg | 1. Waste generation per capita and day:  - < 0,5 kg |
| **PR.6**  [Zero-waste index](#ZWI)  *(nat., reg., mun.)* | 1. Zero-waste index (ZWI):  - ≤ 0,05 ZWI | 1. Zero-waste index (ZWI):  - > 0,05 and ≤ 0,15 ZWI | 1. Zero-waste index (ZWI):  - > 0,15 and ≤ 0,40 ZWI | 1. Zero-waste index (ZWI):  - > 0,40 and ≤ 0,90 ZWI | 1. Zero-waste index (ZWI):  - > 0,90 ZWI |

### Glossary

| Term | Definition |
| --- | --- |
| **Bioeconomy** | „The bioeconomy covers all sectors and systems that rely on biological resources (animals, plants, micro-organisms and derived biomass, including organic waste), their functions and principles. It includes and interlinks: land and marine ecosystems and the services they provide; all primary production sectors that use and produce biological resources, i.e. agriculture, forestry, fisheries and aquaculture; and all economic and industrial sectors that use biological resources and processes to produce food, feed, bio-based products, energy and services. It cuts across these sectors and systems, interlinking them and creating synergies. While biotechnology is at the heart of bio-based processes, health biotechnology and biological medicines are not included in the bioeconomy definition.“ [66] |
| **Circular city** | “A circular city is a city that practices Circular Economy principles to close resource loops, in partnership with the city’s stakeholders (citizens, community, business and knowledge stakeholders), to realize its vision of a future-proof city.” [22] |
| **Circular economy** | A CE is “[…] an economic system that is based on business models which replace the ‘end-of-life’ concept with reducing, alternatively reusing, recycling and recovering materials in production/distribution and consumption processes, thus operating at the micro level (products, companies, consumers), meso level (eco-industrial parks) and macro level (city, region, nation and beyond), with the aim to accomplish sustainable development, which implies creating environmental quality, economic prosperity and social equity, to the benefit of current and future generations.” [67] The idea of CE is to reduce waste to a minimum and keep materials and products as long as possible in the economic cycle [68]. |
| **Circular material use rate (CMUR)** | This “[…] indicator measures the share of material recovered and fed back into the economy - thus saving extraction of primary raw materials - in overall material use. The circular material use (CMU) rate is defined as the ratio of the circular use of materials to the overall material use. The overall material use is measured by summing up the aggregate domestic material consumption (DMC) and the circular use of materials. DMC is defined in economy-wide material flow accounts. The circular use of materials is approximated by the amount of waste recycled in domestic recovery plants minus imported waste destined for recovery plus exported waste destined for recovery abroad. Waste recycled in domestic recovery plants comprises the recovery operations R2 to R11 - as defined in the Waste Framework Directive 75/442/EEC. The imports and exports of waste destined for recycling - i.e. the amount of imported and exported waste bound for recovery – are approximated from the European statistics on international trade in goods. A higher CMU rate value means that more secondary materials substitute for primary raw materials thus reducing the environmental impacts of extracting primary material.“ [69] |
| **Circular supply chain** | Circular supply Chain is defined as “the integration of circular thinking into the management of the supply chains and its surrounding industrial and natural ecosystems. It systematically restores materials toward a zero-waste vision through innovation from product/service design to end-of-life and waste management, involving all stakeholders” [12]. |
| **Closed-loop recycling** | “According to ISO 14044 a loop is closed when “material from a product system is recycled in the same product system. [...] In closed loops, material is recycled without […] changes.” [70] |
| **Collection rate** | “This indicator takes into account all different MSW streams collected separately by all the different collection systems available in the territory considered (e.g. door-to-door, civic amenity sites, street containers). In areas where there is no detailed waste monitoring or where a part of the waste generated is not collected by the formal municipal waste collection system, figures on MSW generation could underestimate the real situation.” [71] |
| **Composting** | “The controlled biological decomposition of organic material in the presence of air to form a humus-like material. Controlled methods of composting include mechanical mixing and aerating, ventilating the materials by dropping them through a vertical series of aerated chambers, or placing the compost in piles out in the open air and mixing it or turning it periodically. [72] |
| **Construction and demolition waste** | “Construction and demolition waste - Rubble and other waste material arising from the construction, demolition, renovation or reconstruction of buildings or parts thereof, whether on the surface or underground. Consists mainly of building material and soil, including excavated soil. Includes waste from all origins and from all economic activity sectors.” [72] |
| **Controlled dump** | “A controlled dump is a non-engineered disposal site where improvement is implemented on the operational and management aspects rather than on facility or structural requirements, which would otherwise require substantial investment. Controlled dumps evolved due to the need to close open dumpsites and replace them with improved disposal facilities, and in consideration of the financial constraints of LGU’s [local government units]. Controlled disposal of wastes may be implemented over existing wastes (from previous open dumping operations) or on new sites.” [73]  According to [54] a controlled dump has the following characteristic:   - *Operation and Engineering Measures:* Registration and placement/compaction of waste; surface water monitoring; no engineering measures - *Leachate Management:* Unrestricted contaminant release - *Landfill Gas Management:* None |
| **Controlled landfill** | “Controlled landfill is a landfill whose operation is subject to a permit system and to technical control procedures in compliance with the national legislation in force. Includes specially engineered landfill.” [72]  According to [54] a controlled landfill/engineered landfill has the following characteristics:   - *Operation and Engineering Measures:* Registration and placement/compaction of waste; uses daily cover material; surface and ground water monitoring; infrastructure and liner in place - *Leachate Management:* Containment and some level of leachate treatment; reduced leachate volume through waste cover - *Landfill Gas Management:* Passive ventilation or flaring |
| **Deposit-refund system** | “Surcharge on the price of potentially polluting products. When pollution is avoided by returning the products or their residuals, a refund of the surcharge is granted.” [72] |
| **Diversion rate** | “Defined as the percentage of total waste that is diverted from disposal at permitted landfills and transformation facilities such as incineration, and instead is directed to reduction, reuse, recycling and composting programs.  $Diversion rate= \frac{Weight of recyclables}{Weight of garbage+Weight of recyclables}*100 \%$ ” [74]  A diversion rate of 0,70 means that 70% of the municipal waste collected is recycled. If technically and economically feasible, the diversion rate can increase to 100%.  The remaining quantities of collected municipal waste are either landfilled or treated thermally or energetically (≤ 30%). |
| **Downcycling** | “Downcycling refers to a recycling process. In contrast to upcycling, downcycling involves a decline in the value of a recyclate, as the quality deteriorates during a progressive number of recycling cycles or more primary raw materials have to be added. Downcycling is particularly important for plastics recycling; it ensures that raw materials continue to be used despite a loss of quality.” [75] |
| **Dual WM** | “To reduce the quantity of packaging waste, and thereby of overall MSW, Germany introduced a far-reaching legislation to reduce waste, based on the producer's responsibility principle. Industry was given the option to set up a third party organization which would carry out the collection and sorting of sales packaging for care of manufacturers and retailers. Thus, some 600 companies created "Duales System Deutschland" in 1990 ("Dual" because it meant creating a second collection system in parallel to the existing waste collection system of the local authorities). Duales System Deutschland (DSD), now has overall responsibility for the separate collection and recycling of packaging. At present, the Dual System is the only nationwide system for the collection and sorting of sales packaging. Packaging participating in this collection system is marked with the Green Dot.” [76] |
| **Eco-design** | “The integration of environmental aspects into the product development process, by balancing ecological and economic requirements. Eco-design considers environmental aspects at all stages of the product development process, striving for products which make the lowest possible environmental impact throughout the product life cycle.“ [72] |
| **Energy recovery** | “A form of resource recovery in which the organic fraction of waste is converted to some form of usable energy. Recovery may be achieved through the combustion of processed or raw refuse to produce steam through the pyrolysis of refuse to produce oil or gas; and through the anaerobic digestion of organic wastes to produce methane gas.” [72] |
| **Engineered landfill** | See controlled landfill. |
| **Extended producer responsibility (EPR)** | “Extended Producer Responsibility (EPR) is a policy approach under which producers are given a significant responsibility – financial and/or physical – for the treatment or disposal of post-consumer products. Assigning such responsibility could in principle provide incentives to prevent wastes at the source, promote product design for the environment and support the achievement of public recycling and materials management goals.” [77] |
| **Hazardous waste** | “The **term “hazardous waste” refers to various types of waste with defined hazardous properties that are harmful for the environment and/or human health. Hazardous waste must be handled using special techniques and processes that ensure safe and environmentally sound disposal by elimination of the hazardous substances contained in the waste.”** [78] |
| **Household waste** | “Solid waste composed of garbage and rubbish, which normally originates from houses.” [72] |
| **Incineration** | “Controlled process by which solid, liquid, or gaseous combustible wastes are burned and changed into gases; residue produced contains little or no combustible material. The aims of the process of burning solid waste under controlled conditions are to reduce its weight and volume, and often to produce energy.” [72] |
| **Informal sector** | “Informal sector waste workers are self-employed and are not officially registered for tax purposes. They do not receive a salary from any organization but derive their income from selling what they pick from the waste or from informal payments from individual households.” [79] |
| **Landfill** | “A waste disposal site for the deposit of the waste onto or into land (i.e. underground).” [72] |
| **Landfill gas** | “Landfill gas means all the gases generated from the landfilled waste.” [72] |
| **Landfill leachate** | “Liquid that has seeped through solid waste in a landfill and has extracted soluble dissolved or suspended materials in the process.” [72] |
| **Life cycle assessment (LCA)** | “Life-cycle assessment (LCA) is a process of evaluating the effects that a product has on the environment over the entire period of its life thereby increasing resource-use efficiency and decreasing liabilities. It can be used to study the environmental impact of either a product or the function the product is designed to perform. LCA is commonly referred to as a "cradle-to-grave" analysis. LCA's key elements are: (1) identify and quantify the environmental loads involved; e.g. the energy and raw materials consumed, the emissions and wastes generated; (2) evaluate the potential environmental impacts of these loads; and (3) assess the options available for reducing these environmental impacts.” [72] |
| **Municipal waste** | “Waste from households, as well as other waste which, because of its nature or composition, is similar to waste from household.” [72] |
| **Open burning** | “"Open burning" is the burning of unwanted materials such as paper, trees, brush, leaves, grass, and other debris where smoke and other emissions are released directly into the air. During open burning, air pollutants do not pass through a chimney or stack.” [80] |
| **Open dump** | “Open dumpsites entail the least development and operational cost requirement […] of land disposal, and thus, are the most prevalent type of disposal facilities in most developing countries. […] they also pose the greatest threat to public health and the environment. […] Open dumpsites are usually located in areas not feasible for such facilities because of the absence of proper siting considerations or criteria. They are usually located in any available vacant area, and are usually within a government-owned property. […] There are no general operational guidelines governing proper operation of the facility and many operators of these dumpsites lack equipment as well as the necessary expertise. Often, burning of waste is done to reduce the volume of waste and preserve disposal space at the site. […] Often, there is no control over the amount and/or type of waste that is disposed of in the site. If wastes other than municipal solid wastes, such as medical and toxic and hazardous wastes, are permitted for disposal in the site, the risks to public health and the environment become more significant. […] Open dumpsites do not have the necessary facilities and measures to control and safely manage liquid and gaseous by-products of waste decomposition.” [73] |
| **Open-loop recycling** | According to ISO 14044 “open loop recycling may, but does not have to, change the inherent properties of the recycled material.” [70] |
| **Organic waste** | “Waste containing carbon compounds.” [72] |
| **Packaging** | “Packaging shall mean all products made of any materials of any nature to be used for the containment, protection, handling, delivery and presentation of goods, from raw materials to processed goods, from the producer to the user or the consumer. Non-returnable items used for the same purposes shall also be considered to constitute packaging. Packaging consists only of:(a) sales packaging or primary packaging, i.e. packaging conceived so as to constitute a sales unit to the final user or consumer at the point of purchase; (b) grouped packaging or secondary packaging, i.e. packaging conceived so as to constitute at the point of purchase a grouping of a certain number of sales units whether the latter is sold as such to the final user or consumer or whether it serves only as a means to replenish the shelves at the point of sale; it can be removed from the product without affecting its characteristics; (c) transport packaging or tertiary packaging, i.e. packaging conceived so as to facilitate handling and transport of a number of sales units or grouped packaging in order to prevent physical handling and transport damage. Transport packaging does not include road, rail, ship and air containers.” [72] |
| **Product-service-systems** | Product service systems that focus on the rental of products and thus provide incentives for low-maintenance, high-quality products with high utility value. This also includes the offer to combine particularly durable products with services for maintenance, repair or upgrades. [7] |
| **Public-private-partnership (PPP)** | “Public-private partnership (PPP) focuses on the privatization of MSW service from the public sector to the private sector. The definition of PPP is “the transfer and control of a good or a service currently provided by the public sector, either in whole or in part, to the private sector. It involves a wide range of private sector participation in public services and serves as a potential strategic management tool.” [44]. [43] and [81] extended the definition of PPP to include formal sectors, informal sectors, private waste contractors, and comparatively formal entities like CBOs (community based organizations) and NGOs (non-governmental organizations).”[82] |
| **Recyclable collection rate (or capture rate)** | “This indicator measures the share of the estimated generation of a specific waste fraction that is collected separately (e.g. plastic, metal, paper and cardboard, glass and co-mingled packaging). This indicator is useful to monitor how efficient is a separate waste collection system in intercepting the recyclable fractions.” [71] |
| **Recycling** | “(1) A resource recovery method involving the collection and treatment of a waste product for use as raw material in the manufacture of the same or a similar product. (2) the EU waste strategy distinguishes between: reuse meant as a material reuse without any structural changes in materials; recycling meant as a material recycling, only, and with a reference to structural changes in products; and recovery meant as an energy recovery only.” [72] |
| **Recycling rates** | “% of waste that is actually recycled or sent for recycling out of the total waste covered by the EPR scheme.” [71]  “Recycling rate for E-waste (in %):  The indicator is calculated by multiplying the 'collection rate' as set out in the WEEE Directive with the 'reuse and recycling rate' set out in the WEEE Directive; where:   - The 'collection rate' equals the volumes collected of WEEE in the reference year divided by the average quantity of electrical and electronic equipment (EEE) put on the market in the previous three years (both expressed in mass unit). - The 'reuse and recycling rate' is calculated by dividing the weight of WEEE that enters the recycling/preparing for reuse facility by the weight of all separately collected WEEE (both in mass unit) in accordance with Article 11(2) of the WEEE Directive 2012/19/EU, considering that the total amount of collected WEEE is sent to treatment/recycling facilities.” [69]   “Recycling rate for construction and demolition waste (in %):  The indicator is the ratio of C&D waste which is prepared for reuse, recycled or subject to material recovery, including through backfilling operations, divided by the C&D waste treated as defined in Regulation (EC) No 2150/2002 on waste statistics. The indicator covers the waste category 'Mineral waste from construction and demolition' (EWC-Stat 12.1). Only non-hazardous waste is taken into account.” [69] |
| **Refuse-derived fuels (RDFs)** | “Refuse derived fuel (RDF) is produced from domestic and business waste, which includes biodegradable material as well as plastics. Non-combustible materials such as glass and metals are removed, and the residual material is then shredded. Refuse derived fuel is used to generate energy at recovery facilities, many of them in Europe where they produce electricity and hot water for communal heating systems.” [83] |
| **Reuse** | “‘Reuse’ means any operation by which products or components that are not waste are used again for the same purpose for which they were conceived.” [84] |
| **Sanitary landfill** | “A sanitary landfill is an engineered disposal facility designed, constructed, and operated in a manner that minimizes impacts to public health and the environment. In contrast to open dumpsites and controlled dumps, sanitary landfills undergo thorough planning right from the selection of the site up to post-closure management.  According to [54] a sanitary landfill has the following characteristic:   - *Operation and Engineering Measures:* Registration and placement/compaction of waste; uses daily cover; measures for final top cover and closure; proper siting, infrastructure; liner and leachate treatment in place and post-closure plan - *Leachate Management:* Containment and leachate treatment (often biological and physico-chemical treatment) - *Landfill Gas Management:* Flaring with or without energy recovery |
| **Separate collection** | “The collection of individual components of solid waste from any source, usually separated into different collection containers, in order to recover, reuse or recycle the material or to facilitate its collection and disposal.” [72] |
| **Stakeholder** | “An institution, organization, or group that has some interest in a particular sector or system.” [72] |
| **Stakeholders in WM** | Stakeholders in WM are: Law and regulation bodies, research and educational institutions, media, WM companies, industry and commerce, service industries, members of the informal sector, planners and consultants in the public and the private sector, non-governmental organizations (NGOs) as well as political parties, associations, societies, trade unions, etc. [29] |
| **Transfer stations** | “Transfer stations play an important role in the overall waste management infrastructure network by providing means of consolidating and managing recyclables and waste. This is particularly important in rural and regional areas where efficient transport logistics determine the overall feasibility of providing waste and recovery services. In its simplest form, a transfer station is a facility with a designated receiving area where waste collection vehicles and/or small self-haul customers discharge their waste. The waste is then loaded into larger vehicles (e.g.: transfer trailers) for longhaul transport to a final disposal site (typically a landfill, treatment facility, materials recovery facility (MRF), or reprocessing plant). Ideally, there is no long-term storage of materials at a transfer station. Waste and recyclables are consolidated and loaded into larger vehicles for movement off site.“ [85] |
| **Upcycling** | “Upcycling is a form of reuse of materials (recycling). Seemingly useless waste products are transformed into new materials with the help of upcycling. Unlike recycling or downcycling, upcycling results in material upgrading. The process of reusing existing materials reduces the need for newly produced raw materials and thus conserves resources.“ [86] |
| **Waste** | “Materials that are not prime products (that is, products produced for the market) for which the generator has no further use in terms of his/her own purposes of production, transformation or consumption, and of which he/she wants to dispose. Wastes may be generated during the extraction of raw materials, the processing of raw materials into intermediate and final products, the consumption of final products, and other human activities. Residuals recycled or reused at the place of generation are excluded.” [72] |
| **Waste bylaw** | A waste bylaw is a municipal ordinance regarding the collection and recovery of household waste at the municipal level concerning matters such as usage and integration into the public system, as well as municipal garbage collection charges. Adapted from [87] |
| **Waste collection** | Periodic or on-demand removal of solid waste from the point of generation, such as specific addresses or designated collection points, to facilities where the waste is recovered or disposed, regardless of collection modality (e.g., by municipal governments, non-state actors or informal sector). (adapted from [72] and [48]). |
| **Waste disposal** | “Disposal means any of the applicable operations provided for in Annex IIA to Directive 91/156/EEC:D1 Tipping above or underground (e.g. landfill, etc.), D2 Land treatment (e.g. biodegradation of liquid or sludge discards in soils, etc.), D3 Deep injection (e.g. injection of pumpable discards into wells, salt domes or naturally occurring repositories, etc.), D4 Surface impoundment (e.g. placement of liquid or sludge discards into pits, ponds or lagoons, etc.), D5 Specially engineered landfill (e.g. placement into lined discrete cells which are capped and isolated from one another and the environment, etc.), D6 Release of solid waste into a water body except seas/oceans, D7 Release into seas/oceans including seabed insertion, D8 Biological treatment not specified elsewhere in this Annex which results in final compounds or mixtures which are disposed of by means of any of the operations in this Annex, D9 Physico-chemical treatment not specified elsewhere in this Annex which results in final compounds or mixtures which are disposed of by means of any of the operations in this Annex (e.g. evaporation, drying, calcination, etc.), D10 Incineration on land, D11 Incineration at sea, D12 Permanent storage (e.g. emplacement of containers in a mine, etc.), D13 Blending or mixture prior to submission to any of the operations in this Annex, D14 Repackaging prior to submission to any of the operations in this Annex, D15 Storage pending any of the operations in this Annex, excluding temporary storage, pending collection, on the site where it is produced.” [72] |
| **Waste dumping** | “The disposal of solid wastes without environmental controls.” [72] |
| **Waste export** | “Transporting unwanted materials, including those leftover from a manufacturing processes, refuse, or trash to other countries or areas for the conduct of foreign trade.” [72] |
| **Waste generation** | “The weight or volume of materials and products that enter the waste stream before recycling, composting, landfilling or combustion takes place. Also can represent the amount of waste generated by a given source or category of sources.” [72] |
| **Waste generation (indicator)** | “The indicator measures the waste collected by or on behalf of municipal authorities and disposed of through the waste management system. It consists to a large extent of waste generated by households, though similar wastes from sources such as commerce, offices and public institutions may be included.” [69] |
| **Waste management (WM)** | “‘Waste management’ means the collection, transport, recovery and disposal of waste, including the supervision of such operations and the after-care of disposal sites, and including actions taken as a dealer or broker;” [84] |
| **Waste management system** | A waste management system (WMS) is a system-relevant infrastructure system, which has the function of collecting, treating (incl. sorting) and disposing all types of generated waste. Activities that promote the waste prevention and reuse or concern trading and broking of waste or secondary raw materials are also part of the system as well as components regarding all governance (e.g. public authorities, laws, regulations), technical (e.g. treatment technologies), organizational (e.g. infrastructure, collecting services), aspects and other resources (e.g. qualified labour, financial resources, natural resources) required for this function. |
| **Waste picker** | “Waste pickers collect household or commercial/industrial waste. They may collect from private waste bins or dumpsters, along streets and waterways or on dumps and landfills. Some rummage in search of necessities; others collect and sell recyclables to middlemen or businesses. Some work in recycling warehouses or recycling plants owned by their cooperatives or associations. The term “waste picker” was adopted at the [First World Conference of Waste Pickers](http://globalrec.org/global-meeting/latin-american-colombia-2008/) in Bogota, Colombia in 2008 to facilitate global networking--and to supplant derogatory terms like “scavenger”. Preferred terms vary, however, by place. For example, [in South Africa](https://www.wiego.org/waste-integration-south-africa-wisa) "reclaimers" and "bagerezi" are used. In the United States, "canners" is often used. Other languages have their own preferred terms: catadores in Portuguese, recicladores in Spanish. What waste pickers have in common is that this work is their livelihood and often helps support their families.” [88] |
| **Waste prevention** | “Prevention’ means measures taken before a substance, material or product has become waste, that reduce: (a) the quantity of waste, including through the reuse of products or the extension of the life span of products; (b) the adverse impacts of the generated waste on the environment and human health; or (c) the content of harmful substances in materials and products” [84] |
| **Waste prevention programs** | “[Waste prevention programmes] shall be integrated either into the waste management plans provided for in [2008/98/EC Directive,] Article 28 or into other environmental policy programmes […] or shall function as separate programmes. If [a] programme is integrated into the waste management plan or into other programmes, the waste prevention measures shall be clearly identified.  The programmes […] shall set out the waste prevention objectives [and] describe the existing prevention measures and indicate the usefulness of the examples of measures indicated […] Appropriate qualitative or quantitative benchmarks for waste prevention measures shall be determined in order to monitor and assess the progress of the measures. Specific qualitative and quantitative targets and indicators for waste prevention measures shall be adopted in accordance with the regulatory procedure.” [84] |
| **Waste recycling** | “‘Recycling’ means any recovery operation by which waste materials are reprocessed into products, materials or substances whether for the original or other purposes. It includes the reprocessing of organic material but does not include energy recovery and the reprocessing into materials that are to be used as fuels or for backfilling operations” [84] |
| **Waste treatment** | “The physical, thermal, chemical or biological processes, which change the characteristics of the waste in order to reduce its volume or hazardous nature, to facilitate its handling or to enhance recovery.” [72] |
| **WM concept** | See also WM plan. |
| **WM plan** | “[A] waste management plan shall set out an analysis of the currents waste management situation in the geographical entity concerned. [It also includes] the measures, that are to be taken to improve environmentally sound preparing for reuse, recycling, recovery and disposal of waste as well as an evaluation of how the plan will support the implementation of the objectives and provisions of [Directive 2008/98/EC].  [A] waste management plan shall contain […]:   - type, quantity and source of waste generated within the territory, the waste likely to be shipped from or to the […] territory, and an evaluation of the development of waste streams in the future; - existing waste collection schemes and major disposal and recovery installations, including […] special arrangements for waste oils, hazardous waste or waste streams addressed by specific legislation; - an assessment of the need for new collection schemes, the closure of existing waste installations, additional waste installation infrastructure […] and the [needed] investments; - sufficient information on the location criteria for site identification and on the capacity of future disposal or major recovery installations, […] - general waste management policies, including planned waste management technologies and methods, or policies for waste posing specific management problems.   [A] waste management plan may contain […]:   - organizational aspects related to waste management including a description of the allocation of responsibilities between public and private actors carrying out the waste management; - an evaluation of the usefulness and suitability of the use of economic and other instruments in tackling various waste problems, taking into account the need to maintain the smooth functioning of the internal market; - the use of awareness campaigns and information provision directed at the general public or at a specific set of consumers; - historical contaminated waste disposal sites and measures for their rehabilitation.   A waste management plan also shall consider the geographical level and coverage of the planning area.” [84]  Waste Management plans, programs or concepts are often used in a same or similar context or even synonymously. |
| **WM program** | See also WM plan. |
| **Zero-waste** | “Zero Waste: The conservation of all resources by means of responsible production, consumption, reuse, and recovery of products, packaging, and materials without burning and with no discharges to land, water, or air that threaten the environment or human health.” [89] |
| **Zero-waste-index** | “The zero waste index is a tool to measure the potentiality of virgin materials to be offset by zero waste management systems. One of the important goals of the zero waste concept is zero depletion of natural resources. Therefore, measuring the performance of the zerowaste city would eventually measure the resources that are extracted, consumed, wasted, recycled, recovered and finally substituted for virgin materials and offset resource extraction by the waste management systems.  The ZWI formula is as follows:  $ZWI=\frac{\sum potential amount of waste management by the city*substitution for the systems}{total amount of waste generated in the city}$  The [ZWI] is based on the value of material that can potentially replace the virgin material inputs. The substitution of energy, water and greenhouse gas emissions is also considered with the material substitutions.” [74] |

**Useful guidelines regarding**:

Waste management (WM) in general: [47,90,91]

Implementation of WM facilities: [92,93]

Implementation of landfills: [73,94,95]

Implementation of incineration plants: [55–57]

Measures for new treatment facilities (Emissions in air, soil and water, occupational and community health and safety): [96]

### References

- - 1. Shekdar, A.V. Sustainable solid waste management: An integrated approach for Asian countries. Waste Manage. 2009, 29, 1438–1448, doi:10.1016/j.wasman.2008.08.025.
    2. Zaman, A.U. A comprehensive review of the development of zero waste management: lessons learned and guidelines. J. Cleaner Prod. 2015, 91, 12–25, doi:10.1016/j.jclepro.2014.12.013.
    3. Silva, A.; Stocker, L.; Mercieca, P.; Rosano, M. The role of policy labels, keywords and framing in transitioning waste policy. J. Cleaner Prod. 2016, 115, 224–237, doi:10.1016/j.jclepro.2015.12.069.
    4. Striegel, K.-H. Stage concept for sustainable circular economy: Contribution of the waste management sector to resource and climate protection within international cooperation. [Stufenkonzept zur nachhaltigen Kreislaufwirtschaft. Beitrag der Abfallwirtschaft zum Ressourcen- und Klimaschutz in der internationalen Zusammenarbeit.], Frankfurt am Main, October 28, 2015.
    5. Schuebeler, P.; Wehrle, K.; Christen, Jürg, SKAT. Conceptual Framework for Municipal Solid Waste Management in Low-Income Countries: Working Paper No. 9, St. Gallen, 1996 (accessed on 20 March 2018).
    6. EC. A new Circular Economy Action Plan: For a cleaner and more competitive Europe. COM(2020) 98 final, Brussels, 2020.
    7. Müller, F.; Kohlmeyer, R.; Krüger, F.; Kosmol, J.; Krause, Susann, Dorer, Conrad; Röhreich, M. Guiding principles of a circular economy [Leitsätze einer Kreislaufwirtschaft], Dessau-Roßlau, 2020 (accessed on 26 November 2020).
    8. Pajunen, N.; Watkins, G.; Husgafvel, R.; Heiskanen, K.; Dahl, O. The challenge to overcome institutional barriers in the development of industrial residue based novel symbiosis products – Experiences from Finnish process industry. Miner. Eng. 2013, 46-47, 144–156, doi:10.1016/j.mineng.2013.03.008.
    9. Schanes, K.; Jäger, J.; Drummond, P. Three Scenario Narratives for a Resource-Efficient and Low-Carbon Europe in 2050. Ecol. Econ. 2019, 155, 70–79, doi:10.1016/j.ecolecon.2018.02.009.
    10. Buschmann, R.; Meyer, K.; Scheider, H.; Heinz, R.; Jedelhauser, M.; Tauer, R. Making the EU fit for Future: What the German environmental associations want from the European Green Deal, 2020 (accessed on 5 February 2021).
    11. Wilson, D.C.; Rodic, L.; Cowing, M.J.; Velis, C.A.; Whiteman, A.D.; Scheinberg, A.; Vilches, R.; Masterson, D.; Stretz, J.; Oelz, B. ‘Wasteaware’ benchmark indicators for integrated sustainable waste management in cities. Waste Manage. 2015, 35, 329–342, doi:10.1016/j.wasman.2014.10.006.
    12. Maranesi, C.; Giovanni, P. de. Modern Circular Economy: Corporate Strategy, Supply Chain, and Industrial Symbiosis. Sustainability 2020, 12, 9383, doi:10.3390/su12229383.
    13. Friant, M.C.; Vermeulen, W.J.; Salomone, R. Analysing European Union circular economy policies: words versus actions. Sustain. Prod. Consum. 2021, Volume 27, p. 337–353. doi:10.1016/j.spc.2020.11.001.
    14. Recycling Magazin. Enforcement and further development of product responsibility [Vollzug und Weiterentwicklung der Produktverantwortung]. Available online: <https://www.recyclingmagazin.de/2017/01/23/vollzug-und-weiterentwicklung-der-produktverantwortung/> (accessed on 7 January 2021).
    15. European Union. Commission Decision of 18th December 2014 - amending Decision 2000/ 532/ EC on the list of waste pursuant to Directive 2008/ 98/ EC of the European Parliament and of the Council -(2014/ 955/ EU): 2014/955/EU. Official Journal of the European Union 2014.
    16. van de Klundert, A.; Anschütz, J. Integrated Sustainable Waste Management - the Concept.: Tools for Decision-makers. Experiences from the Urban Waste Expertise Programme, Gouda, Niederlande, 2001 (accessed on 20 March 2018).
    17. Filho, W.L.; Brandli, L.; Moora, H.; Kruopiene, J.; Stenmarck, A. Benchmarking approaches and methods in the field of urban waste management. J. Cleaner Prod. 2016, 112, 4377–4386, doi:10.1016/j.jclepro.2015.09.065.
    18. Pfaff-Simoneit, W. Development of a sectoral approach for the establishment of sustainable waste management systems in developing countries in the context of climate change and resource scarcity: [Entwicklung eines sektoralen Ansatzes zum Aufbau von nachhaltigen Abfallwirtschaftssystemen in Entwicklungsländern vor dem Hintergrund von Klimawandel und Ressourcenverknappung]. [Dissertation]; Rostock, 2012.
    19. Guerrero, L.A.; Maas, G.; Hogland, W. Solid waste management challenges for cities in developing countries. Waste Manage. 2013, 33, 220–232, doi:10.1016/j.wasman.2012.09.008.
    20. World Bank Group. Solid waste management: optional sectoral module (English)., Washington, D.C., 2018. Available online: <http://documents.worldbank.org/curated/en/733491525783750293/Solid-waste-management-optional-sectoral-module> (accessed on 22 December 2020).
    21. BMUB. Modern waste - Goals and Paths: Germany’s expertise for an advanced circular economy, 2016. Available online: <https://www.giz.de/de/downloads/giz2016-en-vdma_Modern_Waste_Management.pdf> (accessed on 11 January 2022).
    22. Prendeville, S.; Cherim, E.; Bocken, N. Circular Cities: Mapping Six Cities in Transition. Environ. Innov. Soc. Transit. 2018, 26, 171–194, doi:10.1016/j.eist.2017.03.002.
    23. Caniato, M. Assessment and Design of Local Regulation in Solid Waste Management in Low- and Middle-Income Countries. In The Political Economy of Local Regulation: Theoretical Frameworks and International Case Studies; Asquer, A., Becchis, F., Russolillo, D., Eds.; Palgrave Macmillan UK: London, 2017; pp 277–286, ISBN 978-1-137-58828-9.
    24. Fatimah, Y.A.; Govindan, K.; Murniningsih, R.; Setiawan, A. Industry 4.0 based sustainable circular economy approach for smart waste management system to achieve sustainable development goals: A case study of Indonesia. J. Cleaner Prod. 2020, 269, 122263, doi:10.1016/j.jclepro.2020.122263.
    25. Romano, G.; Masserini, L.; Lombardi, G.V. Environmental performance of waste management: Impacts of corruption and public maladministration in Italy. J. Cleaner Prod. 2021, 288, 125521, doi:10.1016/j.jclepro.2020.125521.
    26. Fuss, M.; Vasconcelos Barros, R.T.; Poganietz, W.-R. Designing a framework for municipal solid waste management towards sustainability in emerging economy countries - An application to a case study in Belo Horizonte (Brazil). J. Cleaner Prod. 2018, 178, 655–664, doi:10.1016/j.jclepro.2018.01.051.
    27. European Union. Directive 2010/75/EU of the European Parliament and of the Council of the 24. November 2010 on industrial emissions (integrated pollution prevention and control): 2010/75/EU. (Recast). Official Journal of the European Union 2010.
    28. Geng, Y.; Fu, J.; Sarkis, J.; Xue, B. Towards a national circular economy indicator system in China: an evaluation and critical analysis. J. Cleaner Prod. 2012, 23, 216–224, doi:10.1016/j.jclepro.2011.07.005.
    29. Kranert, M. Introduction to the circular economy [Einführung in die Kreislaufwirtschaft]; Springer Fachmedien Wiesbaden: Wiesbaden, 2017.
    30. Jesus, A. de; Mendonça, S. Lost in Transition? Drivers and Barriers in the Eco-innovation Road to the Circular Economy. Ecol. Econ. 2018, 145, 75–89, doi:10.1016/j.ecolecon.2017.08.001.
    31. Campitelli, A.; Schebek, L. How is the performance of waste management systems assessed globally? A systematic review. J. Cleaner Prod. 2020, 272, doi:10.1016/j.jclepro.2020.122986.
    32. Roidt, M.; Avellán, T. Learning from integrated management approaches to implement the Nexus. J. Environ. Manage. 2019, 237, 609–616, doi:10.1016/j.jenvman.2019.02.106.
    33. ElSaid, S.; Aghezzaf, E.-H. A progress indicator-based assessment guide for integrated municipal solid-waste management systems. J Mater Cycles Waste Manag 2018, 20, 850–863, doi:10.1007/s10163-017-0647-8.
    34. Song, Q.; Li, J.; Zeng, X. Minimizing the increasing solid waste through zero waste strategy. J. Cleaner Prod. 2015, 104, 199–210, doi:10.1016/j.jclepro.2014.08.027.
    35. Bleck, D.; Wettberg, W. Waste collection in developing countries – Tackling occupational safety and health hazards at their source. Waste Manage. 2012, 32, 2009–2017, doi:10.1016/j.wasman.2012.03.025.
    36. Ferronato, N.; Rada, E.C.; Gorritty Portillo, M.A.; Cioca, L.I.; Ragazzi, M.; Torretta, V. Introduction of the circular economy within developing regions: A comparative analysis of advantages and opportunities for waste valorization. J. Environ. Manage. 2019, 230, 366–378, doi:10.1016/j.jenvman.2018.09.095.
    37. Wilts, H.; Berg, H. Digital Circular Economy: The Digital Transformation as a Pioneer of Resource-Saving Material Cycles [Digitale Kreislaufwirtschaft Die Digitale Transformation als Wegbereiter ressourcenschonender Stoffkreisläufe], in: inbrief, Wuppertal Insitute, 2017, 04.
    38. Aleluia, J.; Ferrão, P. Characterization of urban waste management practices in developing Asian countries: A new analytical framework based on waste characteristics and urban dimension. Waste Manage. 2016, 58, 415–429, doi:10.1016/j.wasman.2016.05.008.
    39. Velis, C.A.; Wilson, D.C.; Rocca, O.; Smith, S.R.; Mavropoulos, A.; Cheeseman, C.R. An analytical framework and tool (‘InteRa’) for integrating the informal recycling sector in waste and resource management systems in developing countries. Waste Manage. Res. 2012, 30, 43–66, doi:10.1177/0734242X12454934.
    40. Medina, M. Waste Picker Cooperatives in Developing Countries 2005, doi:10.4324/9780203934074.pt4.
    41. Ezeah, C.; Fazakerley, J.A.; Roberts, C.L. Emerging trends in informal sector recycling in developing and transition countries. Waste Manag. 2013, 33, 2509–2519, doi:10.1016/j.wasman.2013.06.020.
    42. Turcott Cervantes, D.E.; Romero, E.O.; Del Consuelo Hernández Berriel, M.; Martínez, A.L.; Del Consuelo Mañón Salas, M.; Lobo, A. Assessment of some governance aspects in waste management systems: A case study in Mexican municipalities. J. Cleaner Prod. 2021, 278, 123320, doi:10.1016/j.jclepro.2020.123320.
    43. Ahmed, S.A.; Ali, M. Partnerships for solid waste management in developing countries: linking theories to realities. Habitat Int. 2004, 28, 467–479, doi:10.1016/S0197-3975(03)00044-4.
    44. Massoud, M.; El-Fadel, M. Public-private partnerships for solid waste management services. Environ. Manage. 2002, 30, 621–630, doi:10.1007/s00267-002-2715-6.
    45. Hogg, D.; Ballinger, A. The Potential Contribution of Waste Management to a Low Carbon Economy: Main Report, 2015 (accessed on 6 July 2017).
    46. Geng, Y.; Tsuyoshi, F.; Chen, X. Evaluation of innovative municipal solid waste management through urban symbiosis: a case study of Kawasaki. J. Cleaner Prod. 2010, 18, 993–1000, doi:10.1016/j.jclepro.2010.03.003.
    47. World Bank Group. Environmental, Health, and Safety (EHS) Guidelines: General EHS Guidelines: Environmental - Waste Management, 2007 (accessed on 24 November 2020).
    48. UN-Habitat. Waste Wise Cities Tool, 2021 (accessed on 15 December 2021).
    49. Hannan, M.A.; Hossain Lipu, M.S.; Akhtar, M.; Begum, R.A.; Al Mamun, M.A.; Hussain, A.; Mia, M.S.; Basri, H. Solid waste collection optimization objectives, constraints, modeling approaches, and their challenges toward achieving sustainable development goals. J. Cleaner Prod. 2020, 277, 123557, doi:10.1016/j.jclepro.2020.123557.
    50. Ramson, S.R.J.; Moni, D.J.; Vishnu, S.; Anagnostopoulos, T.; Kirubaraj, A.A.; Fan, X. An IoT-based bin level monitoring system for solid waste management. J Mater Cycles Waste Manag 2020, doi:10.1007/s10163-020-01137-9.
    51. Sander, K.; Marscheider-Weidemann, F.; Wilts, H.; Hobohm, J.; Hartfeil, T.; Schöps, D.; Heymann, R. Waste management product responsibility under resource protection aspects (RePro): final report. [Abfallwirtschaftliche Produktverantwortung unter Ressourcenschutzaspekten (RePro)], Texte 52/2019, Dessau-Roßlau, 2019 (accessed on 6 January 2021).
    52. Council Directive 1999/31/EC of 26 April 1999 on the landfill of waste: 1999/31/EC, 1999.
    53. Schodrowski, B. EU Parliament adopts resolution on "New Action Plan for the Circular Economy [EU-Parlament nimmt Entschließung zum „Neuen Aktionsplan für die Kreislaufwirtschaft“ (nCEAP) an]. Press statement, 2021.
    54. World Bank. What a Waste: A Global Review of Solid Waste Management, 2012 (accessed on 14 November 2016).
    55. Rand, T.; Haukohl; J.; Marxen, U. Municipal Solid Waste Incineration: A Decision Maker’s Guide, Washington, D.C., 2000 (accessed on 9 May 2019).
    56. Liu, C.; Nishiyama Toru; Kawamoto, K.; Sasaki, S. Waste-to-Energy Incineration: CCET guideline series on intermediate municipal solid waste treatment technologies Waste-to-Energy Incineration, 2020 (accessed on 16 December 2021).
    57. Mutz, D.; Hengevoss, D.; Hugi, C.; Gross, T. Waste-to-Energy Options in ­Municipal Solid Waste Management: A Guide for Decision Makers in Developing ­ and Emerging Countries, 2017 (accessed on 16 December 2021).
    58. Huysman, S.; Schaepmeester, J. de; Ragaert, K.; Dewulf, J.; Meester, S. de. Performance indicators for a circular economy: A case study on post-industrial plastic waste. Resour. Conserv. Recycl. 2017, 120, 46–54, doi:10.1016/j.resconrec.2017.01.013.
    59. Gundupalli, S.P.; Hait, S.; Thakur, A. A review on automated sorting of source-separated municipal solid waste for recycling. Waste Manag. 2017, 60, 56–74, doi:10.1016/j.wasman.2016.09.015.
    60. Meyer, B.; Lee, R.P. Synthesis gas from waste that is difficult to recycle [Synthesegas aus schwer verwertbaren Abfällen]. UmweltMagazin 2019, 49, 36–39, doi:10.37544/0173-363X-2019-09-36.
    61. 20. requirements for recycling of mineral wastes -technical rules - General part. [20. Anforderungen an die stoffliche Verwertung von mineralischen Abfällen -Technische Regeln - Allgemeiner Teil]: Zuordnungswerte LAGA [Assignment values LAGA], 2003.
    62. Wang, Z.; Li, H.; Yang, X. Vision-based robotic system for on-site construction and demolition waste sorting and recycling. J. Build. Eng. 2020, 32, 101769, doi:10.1016/j.jobe.2020.101769.
    63. Schanes, K.; Dobernig, K.; Gözet, B. Food waste matters - A systematic review of household food waste practices and their policy implications. J. Cleaner Prod. 2018, 182, 978–991, doi:10.1016/j.jclepro.2018.02.030.
    64. Schebek, L.; Kannengießer, J.; Campitelli, A.; Fischer, J.; Abele, E.; Bauerdick, C.; Anderl, R.; Haag, S.; Sauer, A.; Mandel, J.; et al. Resource Efficiency through Industry 4.0 - Potentials for Manufacturing SMEs [Ressourceneffizienz durch Industrie 4.0 - Potenziale für KMU des verarbeitenden Gewerbes], 2017 (accessed on 26 November 2020).
    65. Kaza, S.; Yao, L.; Bhada-Tata, P.; van Woerden, F. What a waste 2.0: A Global Snapshot of Solid Waste Management to 2050, 2018 (accessed on 21 September 2018).
    66. European Commission. A sustainable bioeconomy for Europe: Strengthening the connection between economy, society and the environment: updated bioeconomy strategy, Brüssel, 2018. Available online: <https://op.europa.eu/en/publication-detail/-/publication/edace3e3-e189-11e8-b690-01aa75ed71a1/> (accessed on 17 August 2021).
    67. Kirchherr, J.; Reike, D.; Hekkert, M. Conceptualizing the circular economy: An analysis of 114 definitions. Resour. Conserv. Recycl. 2017, 127, 221–232, doi:10.1016/j.resconrec.2017.09.005.
    68. Korhonen, J.; Nuur, C.; Feldmann, A.; Birkie, S.E. Circular economy as an essentially contested concept. J. Cleaner Prod. 2018, 175, 544–552, doi:10.1016/j.jclepro.2017.12.111.
    69. eurostat. CIRCULAR ECONOMY INDICATORS. Available online: <https://ec.europa.eu/eurostat/web/circular-economy/indicators/monitoring-framework> (accessed on 16 August 2021).
    70. Geyer, R.; Kuczenski, B.; Zink, T.; Henderson, A. Common Misconceptions about Recycling. Journal of Industrial Ecology 2016, 20, 1010–1017, doi:10.1111/jiec.12355.
    71. European Commission. Commission Decision of 3 April 2020 on the sectoral reference document on best environmental management practices, sector environmental performance indicators and benchmarks of excellence for the waste management sector under Regulation (EC) No 1221/2009 on the voluntary participation by organisations in a Community eco-management and audit scheme (EMAS): 2020/519. Decisions. Official Journal of the European Union 2020.
    72. European Environment Agency. EEA Glossary. Available online: <https://www.eea.europa.eu/help/glossary/eea-glossary> (accessed on 16 August 2021).
    73. UNEP. Training Module: Closing an Open Dumpsite and Shifting from Open Dumping to Controlled Dumping and to Sanitary Land Filling, 2005. Available online: <http://wedocs.unep.org/bitstream/handle/20.500.11822/8444/SPC_Training_Module_1.pdf?sequence=3&isAllowed=y> (accessed on 27 June 2017).
    74. Zaman, A.U.; Lehmann, S. The zero waste index: a performance measurement tool for waste management systems in a ‘zero waste city’. J. Cleaner Prod. 2013, 50, 123–132, doi:10.1016/j.jclepro.2012.11.041.
    75. Günther, E. Downcycling: Gabler Wirtschaftslexikon. Available online: <https://wirtschaftslexikon.gabler.de/definition/downcycling-33970> (accessed on 16 August 2021).
    76. GEMET General Multilingual Environmental Thesaurus. Dual waste management. Available online: <https://www.eionet.europa.eu/gemet/en/concept/2365> (accessed on 16 August 2021).
    77. OECD. Extended producer responsibility. Available online: <https://www.oecd.org/env/tools-evaluation/extendedproducerresponsibility.htm> (accessed on 16 August 2021).
    78. Umweltbundesamt. Hazardous Waste. Available online: <https://www.umweltbundesamt.de/en/topics/waste-resources/waste-management/waste-types/hazardous-waste> (accessed on 16 August 2021).
    79. Coad, A. Collection of Municipal Solid Waste: Key issues for Decision-makers in Developing Countries, 2011 (accessed on 14 August 2018).
    80. EGLE Michigan Department of Environment, Great Lakes, and Energy. open burning. Available online: <https://www.michigan.gov/egle/0,9429,7-135-3310_4106_70665_70668---,00.html> (accessed on 16 August 2021).
    81. Oteng-Ababio, M.; Melara Arguello, J.E.; Gabbay, O. Solid waste management in African cities: Sorting the facts from the fads in Accra, Ghana. Habitat Int. 2013, 39, 96–104, doi:10.1016/j.habitatint.2012.10.010.
    82. Ma, J.; Hipel, K.W. Exploring social dimensions of municipal solid waste management around the globe - A systematic literature review. Waste Manage. 2016, 56, 3–12, doi:10.1016/j.wasman.2016.06.041.
    83. Clarity Environmental Limited. what is rdf & srf?: WASTE TO FUEL. Available online: <https://www.clarity.eu.com/waste-to-fuel/what-is-rdf-srf/> (accessed on 16 August 2021).
    84. European Union. Directive 2008/98/EC of the European Parliament and of the Council of 19. November 2008 on waste and repealing certain Directives, 2008.
    85. Department of environment and conservation NSW. Handbook and design of rural and regional transfer stations, Sydney, 2006. Available online: <https://www.epa.nsw.gov.au/-/media/epa/corporate-site/resources/warrlocal/060362-transferstation1.pdf?la=en&hash=FA2C668130BFBA811BFFB532031349EFC4585A47> (accessed on 16 August 2021).
    86. PIUS Info-Portal. Upcycling: Glossar. Available online: <https://www.pius-info.de/service/glossar/upcycling/> (accessed on 16 August 2021).
    87. Umweltbundesamt. Waste regulations: Legal framework. Available online: <https://www.umweltbundesamt.de/en/topics/waste-resources/waste-management/waste-regulations> (accessed on 16 August 2021).
    88. WIEGO. Waste Pickers. Available online: <https://www.wiego.org/informal-economy/occupational-groups/waste-pickers>.
    89. ZWIA. ZW Definition. Available online: <http://zwia.org/standards/zw-definition/> (accessed on 7 September 2018).
    90. Bilitewski, B.; Wagner, J.; Reichenbach, J. Best Practice Municipal Waste Management: Information pool on approaches towards a sustainable design of municipal waste management and supporting technologies and equipment. Texte 40/2018, 2018 (accessed on 30 August 2018).
    91. Kobus, D. Practical Guidebook on Strategic Planning in Municipal Waste Management: A Knowledge Product of the Cities of Change, Gütersloh, Washington, D.C., 2003.
    92. World Bank Group. Environmental, Health, and Safety Guidelines for Waste Management Facilities, 2007 (accessed on 24 November 2020).
    93. Kawai, K.; Liu, C.; Premakumara, J.D.G. Composting: CCET guideline series on intermediate municipal solid waste treatment technologies, 2020 (accessed on 16 December 2021).
    94. Rushbrook, P.; Pugh, M. Solid Waste Landfills in Middle- and Lower Income countries: A Technical Guide to Planning, Design and Operation, 1999 (accessed on 6 May 2021).
    95. International Solid Waste Association. A Roadmap for closing Waste Dumpsites: The World’s most Polluted Places.
    96. World Bank Group. Environmental, Health, and Safety General Guidelines: General EHS Guidelines: Introduction, 2007 (accessed on 6 November 2020).

1. All political levels means on national, regional (subnational) and municipal level. [↑](#footnote-ref-2)
2. Waste law describes at minimum: scope, WM tasks, relevant WM stakeholders, and actions for achieving the defined targets. [↑](#footnote-ref-3)
3. European Waste Framework Directive (<https://eur-lex.europa.eu/eli/dir/2008/98>) and its updates (<https://eur-lex.europa.eu/legal-content/EN/TXT/?uri=CELEX%3A02008L0098-20180705&qid=1642604892683>) as orientation. When implementing laws based on EU standards (<https://ec.europa.eu/environment/topics/waste-and-recycling/waste-law_en>), it is important to adapt the laws to local conditions. [↑](#footnote-ref-4)
4. European directives with a focus on waste streams (e.g., biowaste, waste electronics, packaging, batteries, sewage sludge, vehicles, hazardous substances, commercial waste) as orientation: <https://ec.europa.eu/environment/waste/legislation/c.htm>. [↑](#footnote-ref-5)
5. European directives related to WM facilities (e.g., landfills, waste incineration plants) as orientation: <https://ec.europa.eu/environment/waste/legislation/b.htm>. [↑](#footnote-ref-6)
6. Laws promoting material recycling, eco-design, and the reparability and longevity of products [6]. Guiding legal regulations (e.g., to adjust the economic framework conditions or target quotas) are implemented if other drivers in the market are lacking or are insufficient [7]. [↑](#footnote-ref-7)
7. This right obliges manufacturers to make their products repairable and to provide spare parts, software updates and repair manuals, volunteer repair initiatives and consumers for at least a specified period of time [10],[6]. [↑](#footnote-ref-8)
8. E.g., tax relief, increase of statutory warranty depending on product, repair index (see also <https://www.ecologie.gouv.fr/indice-reparabilite>). [↑](#footnote-ref-9)
9. E.g., commercial, transport, regulatory, customs and environmental law. [↑](#footnote-ref-10)
10. These include global agreements such as: Basel Convention (1989), London Convention (1972), Joint Convention (Vienna, 1997), Hong Kong International Convention (2009), Kyoto Protocol incl. amendments (1997) or Paris Agreement (2016), Espoo Convention (1997), Aarhus Convention (1998), Nairobi (2007) and agreements for specific associations of states, such as Bamako (1991; for African states), Waigani (1995, South Pacific states), Decision of the Council on the Control of Transboundary Movements of Wastes Destined for Recovery Operations (2001, for OECD states). [↑](#footnote-ref-11)
11. Manufacturers must carry out cleaning measures themselves or bear the costs in the event of improper behavior by users. [↑](#footnote-ref-12)
12. European Waste Catalogue [15] for orientation. [↑](#footnote-ref-13)
13. “Regional” means political structures and institutions that lie between the municipal level and the national level. These can vary from nation to nation. If no regional structures are existent, then the points mentioned here should exist either at national or municipal level. [↑](#footnote-ref-14)
14. E.g., by a functioning fee collection system or by increased support for higher levels of administration. [↑](#footnote-ref-15)
15. E.g., joint waste collection, realization and maintenance of WM facilities. [↑](#footnote-ref-16)
16. E.g., through a functioning purpose-based socially responsible fee collection (e.g., consumption- or demand-based contributions) or increased promotion of higher administrative levels. Increase of willingness to pay, waste fees are linked to other taxes/charges (e.g. property, energy or water) [17],[18]. [↑](#footnote-ref-17)
17. E.g., inspections, reporting requirements, registration and notification requirements. [↑](#footnote-ref-18)
18. See also European Union emission limit values for emissions from industry (incl. emissions from waste treatment plants) [27]. [↑](#footnote-ref-19)
19. Different indicators are used on the different levels, which makes a benchmark impossible [28]. [↑](#footnote-ref-20)
20. In the case of small regions (island states or small countries), there are may be no universities. If this is the case, it should be checked whether there are cooperation’s with other countries that enable the training of qualified personnel and research in WM. [↑](#footnote-ref-21)
21. Applies to small states or island states. [↑](#footnote-ref-22)
22. E.g. campaigns on proper waste separation, recycling and prevention of wild dumping, increasing the acceptance of waste treatment facilities, and on environmental and climate protection (e.g., clean-up campaigns). [↑](#footnote-ref-23)
23. Among these measures are: uniforms, gloves, low loading heights, risk allowances, medical insurance, health care, etc. [16]. [↑](#footnote-ref-24)
24. E.g., by impairing access to wastes [39]. [↑](#footnote-ref-25)
25. Possible measures to integrate the informal sector into the formal sector, see the catalog of measures in [39]. [↑](#footnote-ref-26)
26. E.g., measures to increase energy and resource efficiency as well as measures for low-waste, low-emission or low-pollution production and the recycling of waste from production. [↑](#footnote-ref-27)
27. E.g., through better coordination between different public institutions and stricter monitoring of the allocation of funding. [↑](#footnote-ref-28)
28. E.g., packaging, electronic equipment, batteries, end-of-life vehicles. [↑](#footnote-ref-29)
29. Also, in combination with data warehouses and big data applications. [↑](#footnote-ref-30)
30. E.g., audits to improve quality management (ISO 9001) and environmental management (ISO 14001), to increase occupational health and safety (according to ISO 45001) and energy efficiency (ISO 5001). [↑](#footnote-ref-31)
31. E.g., compost, waste paper, waste glass, metal, [RDFs](#RDF). [↑](#footnote-ref-32)
32. E.g., used cars, cell phones, notebooks, TV’s. [↑](#footnote-ref-33)
33. Through e.g. the Internet of Things, recyclable products can market themselves on platforms by publishing relevant product information, such as composition and possible applications [37]. [↑](#footnote-ref-34)
34. E.g., places of interest, with high visibility, culturally or politically important areas as well as wealthy areas and for companies who are willing to pay. [↑](#footnote-ref-35)
35. Depending on the waste material, climate conditions, container type or region, this can vary from daily to monthly. [↑](#footnote-ref-36)
36. Underground systems reduce emissions (noise, pollution) and contribute to a more attractive cityscape than aboveground systems. [↑](#footnote-ref-37)
37. Possible measures e.g., data collection during waste collection (through e.g. QR coding), sensor-based waste containers [50], GPS for more efficient route planning [49]. [↑](#footnote-ref-38)
38. Reasons for this are: systematic waste separation, manual misthrow control by collection service providers and a growing separation awareness among the population. [↑](#footnote-ref-39)
39. Metals include ferrous and non-ferrous metals; paper includes cardboard; plastics includes composites. [↑](#footnote-ref-40)
40. For example, for the collection of organic waste, vehicles with rotary drum can be used to homogenize the waste. [↑](#footnote-ref-41)
41. Landfill classes: landfill for hazardous waste, landfill for non-hazardous waste and landfill for inert waste [52]. [↑](#footnote-ref-42)
42. Depending on the type of waste, the following incineration technologies are used: Residual waste: grate firing; sewage sludge: fluidized bed firing; hazardous waste: rotary kiln. Further thermal utilization technologies are pyrolysis and gasification. [↑](#footnote-ref-43)
43. Incineration plants do not necessarily have to be physically present in the investigated city. In the case of very small municipalities, regions, countries or island states, they may well be located in a neighbouring country and be jointly used by the city under investigation or the waste may be recycled there. [↑](#footnote-ref-44)
44. Measures as cyber-physical systems, block chain applications, digital passports. [↑](#footnote-ref-45)
45. Possible measures to avoid food waste see [63]. [↑](#footnote-ref-46)
46. The use of disposables is banned or only permitted in certain exceptions.E.g., the use of disposables to ensure sterility of products or in cases where the majority of the disposable product is made of recyclates or biobased materials. [↑](#footnote-ref-47)
47. E.g., identify potentials of waste avoidance, recycling of materials and increase of product output and e.g. ensure that recycled materials/wastes are returned in the process or sold to other costumers. [↑](#footnote-ref-48)
48. Orientation towards waste generation index [65]. [↑](#footnote-ref-49)
