# Supplementary material for: Approach to assess the performance of waste management systems towards a circular economy: waste management system development stage concept (WMS-DSC)
Source: MethodsX. 2022 Feb 17;9:101634. doi: 10.1016/j.mex.2022.101634 (PMC8886049; doi:10.1016/j.mex.2022.101634)
Supplement: Supplementary file 3 — S3: Overview of used SDGs and their assigned WMS-DSC subcomponents [file mmc3.docx]

**S3:** Overview of used SDGs and their assigned WMS-DSC subcomponents

| **UN Sustainable Development Goals (SDG’s) and their indicators** | | **WMS-DSC subcomponents** |
| --- | --- | --- |
| **Goal 3. Ensure healthy lives and promote well-being for all at all ages** | | |
|  | 3.3 By 2030, end the epidemics of AIDS, tuberculosis, malaria and neglected tropical diseases and combat hepatitis, water-borne diseases and other communicable diseases | **G.15** Occupational health and safety |
|  | 3.8 Achieve universal health coverage, including financial risk protection, access to quality essential health-care services and access to safe, effective, quality and affordable essential medicines and vaccines for all |  |
| **Goal 4. Ensure inclusive and equitable education and promote lifelong learning opportunities for all** | | |
|  | 4.7 By 2030, ensure that all learners acquire the knowledge and skills needed to promote sustainable development, including, among others, through education for sustainable development and sustainable lifestyles, human rights, gender equality, promotion of a culture of peace and non-violence, global citizenship and appreciation of cultural diversity and of culture’s contribution to sustainable development | **G.12** Education |
| **Goal 6. Ensure available and sustainable management of water and sanitation for all** | | |
|  | 6.3 By 2030, improve water quality by reducing pollution, eliminating dumping and minimizing release of hazardous chemicals and materials, halving the proportion of untreated wastewater and substantially increasing recycling and safe reuse globally | **CT. 8** Collection of hazardous waste  **WD.1** Waste disposal  **WD.3** Leachate water management |
| **Goal 7. Ensure access to affordable, reliable, sustainable and modern energy for all** | | |
|  | 7.1 By 2030, ensure universal access to affordable, reliable and modern energy services | **ER.3** Energy and raw material recovery |
|  | 7.2 By 2030, increase substantially the share of renewable energy in the global energy mix | **ER.3** Energy and raw material recovery |
| **Goal 8. Promote sustained, inclusive and sustainable economic growth, full and productive employment and decent work for all** | | |
|  | 8.3 Promote development-oriented policies that support productive activities, decent job creation, entrepreneurship, creativity and innovation, and encourage the formalization and growth of micro-, small- and medium-sized enterprises, including through access to financial services | **SM.3** Informal sector |
|  | 8.4 Improve progressively, through 2030, global resource efficiency in consumption and production and endeavour to decouple economic growth from environmental degradation, in accordance with the 10-Year Framework of Programmes on Sustainable Consumption and Production, with developed countries taking the lead | **WR.8** Diversion rate and circular material use rate  **PR.1** Prevention |
|  | 8.8 Protect labour rights and promote safe and secure working environments for all workers, including migrant workers, in particular women migrants, and those in precarious employment | **G.15** Occupational health and safety |
| **Goal 9. Build resilient infrastructure, promote inclusive and sustainable industrialization and foster innovation** | | |
|  | 9.4 By 2030, upgrade infrastructure and retrofit industries to make them sustainable, with increased resource-use efficiency and greater adoption of clean and environmentally sound technologies and industrial processes, with all countries taking action in accordance with their respective capabilities | **SM.6** Enterprises |
|  | 9.5 Enhance scientific research, upgrade the technological capabilities of industrial sectors in all countries, in particular developing countries, including, by 2030, encouraging innovation and substantially increasing the number of research and development workers per 1 million people and public and private research and development spending | **G.13** Research |
|  | 9.a Facilitate sustainable and resilient infrastructure development in developing countries through enhanced financial, technological and technical support to African countries, least developed countries, landlocked developing countries and small island developing States |  |
| **Goal 10. Reduce inequality within and among countries** | | |
|  | 10.5 Improve the regulation and monitoring of global financial markets and institutions and strengthen the implementation of such regulations | **G.8** Control mechanisms |
| **Goal 11. Make cities and human settlements inclusive, safe, resilient and sustainable** | | |
|  | 11.6 By 2030, reduce the adverse per capita environmental impact of cities, including by paying special attention to air quality and municipal and other waste management  11.6.1 Proportion of municipal solid waste collected and managed in controlled facilities out of total municipal waste generated, by cities | **CT.3** Collection rates  **WD.1** Waste disposal |
|  | 11.b By 2020, substantially increase the number of cities and human settlements adopting and implementing integrated policies and plans towards inclusion, resource efficiency, mitigation and adaptation to climate change, resilience to disasters, and develop and implement, in line with the Sendai Framework for Disaster Risk Reduction 2015–2030, holistic disaster risk management at all levels | **G.5** Regional level waste management  **G.6** Municipal level waste management |
| **Goal 12. Ensure sustainable consumption and production patterns** | | |
|  | 12.1 Implement the 10-Year Framework of Programmes on Sustainable Consumption and Production Patterns, all countries taking action, with developed countries taking the lead, taking into account | **G.2** Laws, Regulations and Agreements |
|  | 12.2 By 2030, achieve the sustainable management and efficient use of natural resources | **WR.8** Diversion rate and circular material use rate  **PR.1** Prevention |
|  | 12.4 By 2020, achieve the environmentally sound management of chemicals and all wastes throughout their life cycle, in accordance with agreed international frameworks, and significantly reduce their release to air, water and soil in order to minimize their adverse impacts on human health and the environment  12.4.1 Number of parties to international multilateral environmental agreements on hazardous waste, and other chemicals that meet their commitments and obligations in transmitting information as required by each relevant agreement  12.4.2 (a) Hazardous waste generated per capita; and (b) proportion of hazardous waste treated, by type of treatment | **G.2** Laws, Regulations and Agreements  **CT.8** Collection of hazardous waste  **WR.4** Sorting and recycling plants  **PR.1** Prevention |
|  | 12.5 By 2030, substantially reduce waste generation through prevention, reduction, recycling and reuse  12.5.1 National recycling rate, tons of material recycled | **PR.5** Waste generation  **WR.8** Diversion rate and circular material use rate  **PR.5** Waste generation |
|  | 12.6 Encourage companies, especially large and transnational companies, to adopt sustainable practices and to integrate sustainability information into their reporting cycle  12.6.1 Number of companies publishing sustainability reports | **SM.6** Enterprises |
|  | 12.7 Promote public procurement practices that are sustainable, in accordance with national policies and priorities | **G.2** Laws, Regulations and Agreements |
|  | 12.8 By 2030, ensure that people everywhere have the relevant information and awareness for sustainable development and lifestyles in harmony with nature | **G.12** Education  **G.14** Awareness building |
|  | 12.c Rationalize inefficient fossil-fuel subsidies that encourage wasteful consumption by removing market distortions, in accordance with national circumstances, including by restructuring taxation and phasing out those harmful subsidies, where they exist, to reflect their environmental impacts, taking fully into account the specific needs and conditions of developing countries and minimizing the possible adverse impacts on their development in a manner | **G.2** Laws, Regulations and Agreements |
